# piR-26441 inhibits mitochondrial oxidative phosphorylation and tumorigenesis in ovarian cancer through m6A modification by interacting with YTHDC1

**DOI:** 10.1038/s41419-025-07340-6

**Published:** 2025-01-18

**Authors:** Jing Yuan, Bu-Min Xie, Yu-Meng Ji, Hai-Juan Bao, Jie-Lin Wang, Jia-Chen Cheng, Xiang-Chun Huang, Yang Zhao, Shuo Chen

**Affiliations:** Department of Obstetrics and Gynecology, Department of Gynecologic Oncology Research Office; Guangzhou Key Laboratory of Targeted Therapy for Gynecologic Oncology; Guangdong Provincial Key Laboratory of Major Obstetric Diseases; Guangdong Provincial Clinical Research Center for Obstetrics and Gynecology; Guangdong-Hong Kong-Macao Greater Bay Area Higher Education Joint Laboratory of Maternal-Fetal Medicine; The Third Affiliated Hospital, Guangzhou Medical University, Guangzhou, China

**Keywords:** Cancer, Molecular biology

## Abstract

Ovarian cancer (OC) is a heterogeneous cancer. In contrast to other tumor cells, which rely primarily on aerobic glycolysis (Warburg effect) as their energy source, oxidative phosphorylation (OXPHOS) is also one of its major metabolic modes. Piwi-interacting RNAs (piRNAs) play a regulatory function in various biological processes in tumor cells. However, the role and mechanisms of piRNAs in OC and mitochondrial OXPHOS remain to be elucidated. Here, we found that piR-26441 was aberrantly downregulated in OC, and its overexpression suppressed the malignant features of OC cells and tumor growth in a xenograft model. Moreover, overexpression of piR-26441 significantly reduced mitochondrial OXPHOS levels in OC cells. Furthermore, piR-26441 directly binds to and upregulates the expression of YTHDC1 in OC cells. piR-26441 also increased m6A levels, thereby interacting with YTHDC1 to destabilize the mRNA of TSFM. The resultant TSFM loss reduced mitochondrial complex I activity and mitochondrial OXPHOS, leading to mitochondrial dysfunction in OC cells, increased reactive oxygen species levels, and thus, DNA damage and apoptosis in OC cells, thereby inhibiting OC progression. Additionally, ago-piR-26441 suppressed tumor growth and mitochondrial metabolism in the patient-derived organoid model. Altogether, piR-26441 could inhibit OC cell growth via the YTHDC1/TSFM signaling axis, underscoring its significant importance in the context of OC, as well as offering potential as a therapeutic target.

## Introduction

Ovarian cancer (OC) is a common malignant cancer with the highest death rate among females in the world [1]. In 2020, an estimated 313,959 new cases of OC and 207,252 OC-related deaths were reported worldwide [2]. Presently, the treatment strategy for OC primarily depends on the clinical pathology assessment of the tumor, followed by surgery and then chemotherapy using platinum-based drugs and taxanes. However, despite notable advances in surgery and chemotherapy, the survival rate for patients remains low compared with that of other gynecological cancers. This disparity arises from the asymptomatic nature of early-stage OC, the absence of effective biomarkers for early detection, and consequently, poor prognosis and high recurrence rates among patients [3–5]. Hence, exploring molecular mechanisms underlying OC occurrence and progression is crucial for developing effective treatment strategies.

P-element induced wimpy testis (PIWI)-interacting RNAs (piRNAs) belong to a class of 24–31 nucleotides (nt)-long small endogenous non-coding RNAs, first discovered in germ cells [6]. piRNAs combine with PIWI proteins to form piRNAs/PIWI complexes, thereby affecting protein, genome rearrangement, spermatogenesis, epigenetic regulation, and reproductive stem cell maintenance. Reportedly, piRNAs regulate gene expression in cancer in the following three ways: transcriptional gene silencing (TGS), post-TGS (PTGS), and protein interaction [7]. For example, the interaction between piR-823 and heat-shock factor 1 (HSF1) has been demonstrated to facilitate HSF1 activation and Ser326 phosphorylation, resulting in increased colorectal cancer cell proliferation [8].

Among the chemical modifications of RNA, N6-methyladenosine (m6A) is a common, abundant and conserved internal transcription modification in various RNAs [9]. m6A modification has been implicated in the onset and development of several types of cancers by regulating the maturation, localization, translation, and metabolism of RNA. Reportedly, three main methylation regulators, namely, methylation writers, erasers, and readers, regulate the biological effects of m6A. Notable differences have been reported in m6A modification levels in tumor tissues, and it has been closely related to the development and prognosis of tumors. For example, piRNA-30473 has been shown to downregulate Wilms’ tumor 1-associating protein (WTAP) expression via TGS, thereby regulating hexokinase 2 to promote the progression of diffuse large B cell lymphoma [10].

Metabolic reprogramming refers to the adjusting of metabolic processes of cells according to changing environmental conditions in response to stress and increased energy demands, and it has been defined as a essential feature of human cancers [11, 12]. To meet their energy needs, tumor cells have been reported to give preference to glycolysis, even under the condition of sufficient oxygen, which is consistent with the Warburg effect [13]. Recent studies show that the metabolism of tumor cells exhibits heterogeneity, with tumor subgroups preferring typical Warburg effect (aerobic glycolysis) or OXPHOS (oxidative phosphorylation) [14–17]. In OC, tumor cells are divided into subpopulations according to OXPHOS, favoring either OXPHOS (dependent on the tricarboxylic acid cycle) or glycolysis [18]. Interestingly, Huang et al. reported that the piRNA-mediated silencing of transposable elements in both the fly and mouse germ lines required zuc/MitoPLD activity [19]. Altogether, the interaction of mitochondria with the piRNA pathway indicates the possible role of piRNAs in cancer metabolism regarding energy metabolism.

The point of this study was to investigate the role and mechanisms of piRNAs in OC. Herein, we detected low expression of piR-26441 in OC, which inhibits malignant progression of OC cells in vitro and in vivo. Mechanistically, piR-26441 interacted with the m6A regulator YTH m6A RNA-binding protein C1 (YTHDC1) to disrupt the messenger RNA (mRNA) stability of the Ts translation elongation factor, mitochondrial (TSFM), leading to mitochondrial dysfunction, thereby inhibiting mitochondrial respiration and activating reactive oxygen species (ROS) in OC cells. Excessive ROS levels resulted in DNA damage and initiation of apoptosis, suppressing cancer progression. Altogether, the findings revealed that piR-26441 overexpression inhibits mitochondrial respiration and tumorigenesis in OC, providing implications for targeted therapy.

## Materials and methods

### OC specimens

The specimens of OC tissues (*n* = 80) and normal ovary (*n* = 18) were taken from the Third Affiliated Hospital of Guangzhou Medical University. The study protocol was approved by the ethics committee of the Third Affiliated Hospital of Guangzhou Medical University. Written informed consent was given by all patients before participating.

### Cell culture and RNA transfection

The human OC cell lines A2780, CAOV3, HO8910 and OVCAR3 were obtained from Jennio Biotech (Guangzhou, China) and American Type Culture Collection (Manassas, VA, USA). For RNA transfection, Lipofectamine 2000 was used according to the instructions of the manufacturer (Invitrogen, Carlsbad, CA, USA). piR-26441-mimicking siYTHDC1 and negative control were synthesized by Ribobio (Guangzhou, China). Additionally, the short hairpin (sh)RNA for piR-26441 and the pCDNA3.1(+) vector for TSFM were designed and synthesized by Han Heng Company (China).

### Small RNA (sRNA) sequencing (sRNA-Seq)

sRNA-Seq using HiSeq 2500 (Illumina, USA) was performed by Guangzhou Ribobio Co. (China), as previously described [20].

### In vitro cell vitality and migration assays

Cell viability and migration were assessed using the Cell Counting Kit (CCK)-8 assay and a scratch assay, respectively, as previously described [21].

### Apoptosis assay

As previously described [21], apoptosis was determined by using propidium iodide (PI) and fluorescein isothiocyanate (FITC)-Annexin V (BD Pharmingen, San Diego, CA, USA).

### Quantitative real-time reverse transcription PCR (qRT-PCR) and RNA stability assay

As previously described [21], the total RNA was extracted using the TRIzol reagent (Takara, Shiga, Japan) and reverse transcribed to complementary DNA. Next, quantitative real-time PCR was performed.

To assess the RNA stability, cells were inoculated into six-well plates and transfected with the appropriate constructs; cells were incubated until 70% confluence was reached. Next, actinomycin D (ActD; 5 μg/mL; Sigma, St. Louis, MO, USA) was added, and cells were harvested at 0, 1, 2, and 3 h, and TSFM mRNA levels were detected by qRT-PCR.

### Co-immunoprecipitation (Co-IP) and western blot assay

The extraction of proteins was conducted using the radioimmunoprecipitation assay (RIPA) lysis buffer (Beyotime, Shanghai, China). For Co-IP, the proteins from OC cells and rabbit YTHDC1 antibody (Proteintech) or normal rabbit immunoglobulin (Ig)G (negative control) and A + G beads (Beyotime, China) were incubated at 4 °C for 12 h. Following this, the beads were washed six times on ice with 1× RIPA buffer. Finally, the beads were boiled to obtain the sample buffer, which was then subjected to western blotting.

For western blotting, proteins were separated by sodium dodecyl sulfate-polyacrylamide gel electrophoresis and immunoblotted using primary and secondary antibodies. The following antibodies were used: rabbit anti-YTHDC1 (1:5000, Proteintech), rabbit anti-TSFM (1:1000, Proteintech), rabbit anti-nicotinamide adenine dinucleotide+hydrogen:ubiquinone oxidoreductase subunit B8 (NDUFB8; 1:5000, Proteintech), anti-tripartite motif containing 56 (TRIM56; 1:1000, Zenbio), anti-glyceraldehyde-3-phosphate dehydrogenase (GAPDH; 1:5000, Proteintech), and anti-β-actin (1:2000, Proteintech).

### RNA-binding protein immunoprecipitation (RIP) and methylated RIP (meRIP) sequencing

Briefly, cells were lysed using the RIP lysis buffer containing RNA and proteases inhibitors. The cell extracts were incubated with magnetic beads and anti-rabbit YTHDC1 (Proteintech) or normal rabbit IgG (negative control) in the RIP lysis buffer. Subsequently, the binding complexes were washed, purified, and subjected to qRT-PCR.

As previously described [22], the RNA samples were incubated with m6A antibody-conjugated microbeads in the immunoprecipitation buffer overnight at 4 °C. Next, m6A-containing RNA samples were then removed from the magnetic beads. The meRIP sequencing was performed using HiSeq 2500 (Illumina, USA) at Guangzhou Epibiotek Co. (China).

### Fluorescence in situ hybridization (FISH)

FISH was performed following the instructions of the Fluorescence In Situ Hybridization Kit (RiboBio, Guangzhou), as described previously [21]. The reverse probe for piR-26441 (CCUCAGAGUUCCAGACACGUAUUCAUUGUC) was synthesized by Sangon Biotech.

### RNA pulldown assay

Bioprobes for piR-26441 (GACAATGAATACGTGTCTGGAACTCTGAGG) and its antisense were synthesized by Sangon company. Subsequently, pulldown experiments were performed using an RNA pulldown kit (BersinBio), following the protocol of the manufacturer.

### Ubiquitin determination

To determine ubiquitin levels, cells were transfected with piR-26441 mimic and treated with MG132 for 6 h. Cell lysates were then incubated with anti-YTHDC1 antibody at 4 °C overnight. The next steps were the same as for Co-IP. Western blotting was performed to detect the changes in ubiquitin levels.

### Dot blot of m6A

Poly(A) RNA was purified from the total cellular RNA using the Dynabeads mRNA Purification Kit (ThermoFisher), denatured at 95 °C, and spotted onto a nylon membrane. The membranes were blocked with 5% bovine skimmed milk for 1 h after ultraviolet cross-linking, followed by immunoblotting with primary and secondary antibodies. The BIO-RAD imaging system was used for visualization. Additionally, the poly(A) RNA sample was spotted onto a fresh membrane, stained with 0.02% methylene blue in 0.3 M sodium acetate (pH 5.2) for 1 h, and visualized.

### Mitochondrial energy metabolism analysis

The cellular oxygen consumption rate and extracellular acidification rate were measured using oxygen consumption rate assay kit (R01 fluorescent probe method, BBcellProbe®) and Extracellular acidification Rate Assay Kit (BBcellProbe®), following the instructions of the manufacturer.

### Mitochondrial membrane potential assay

Mitochondrial membrane potential was measured using the Mitochondrial Membrane Potential Assay Kit (JC-1, Beyotime), following the instructions of the manufacturer.

### Mitochondrial isocitrate dehydrogenase (IDH) activity assay

Mitochondrial isocitrate dehydrogenase activity was measured using Mitochondrial isocitrate dehydrogenase (ICDHm) activity assay kit (Solarbio, guangzhou), following the instructions of the manufacturer.

### Intracellular lipid quantification

Intracellular lipids were quantified using Nile Red reagent (Macklin), following the instructions of the manufacturer.

### ROS assay

The oxidation-sensitive fluorescent dye 2’,7’-dichlorodihydrofluorescein diacetate (DCFH-DA; Beyotime, Shanghai, China) was used to detect the intracellular ROS levels, following the instructions of the manufacturer.

### DNA damage assay

Cells were examined for DNA damage using the Comet Electrophoresis Kit (Beyotime, Shanghai, China), following the instructions of the manufacturer.

### OC organoids

As previously described [23], OC organoids were generated. Briefly, OC tissues were cut into 1–3 mm pieces and digested with a digestive solution [24] for 60 min. The organoids were suspended in a mixture of 40% complete medium [25] and 60% Matrigel. In a pre-warmed 48-well plate (Corning Petri dish), 45 µL droplets of the medium were placed and passaged every 10–15 d. Next, 50 nmol/L ago-piR-26441 was added to the medium, and the organoids were further co-incubated for 5 d. Images were acquired using an inverted microscope (Nikon) with 4× magnification to observe the proliferation of the organoids.

### Nude mouse xenograft experiment

All animal experiments were performed according to National Institutes of Health guidelines for laboratory animals and approved by Guangdong Medical Laboratory Animal Center. In order to develop a xenograft model, 12 nude mice (1 × 10^7^cells per 200 μL of FBS-free medium) were injected subcutaneously with CAOV3 cells. Once the tumour volumes had reached a diameter of 5 mm, the mice were randomly assigned to one of two treatment groups, namely ago-piR-26441 and control groups, and subcutaneously administered with ago-piR-26441 and control treatment, respectively. Tumors were measured twice a week, and the tumor volume was calculated as follows: Tumor volume = (length×width^2^)/2

At the conclusion of the experiment or upon the mice’s demise, the tumours were excised from the euthanised or dead mice, respectively.

### Statistical analyses

The GraphPad Prism software (version 8.02 [263]) was used for statistical analyses. The nonparametric Mann–Whitney test was performed for small samples that did not fit a normal distribution, and the Student’s t-test was performed for normally distributed large samples. Pearson’s correlation coefficient was used to determine the correlation among different groups. Data from the CCK-8 assay were analyzed with variance analysis. All data are given as mean ± standard deviation, and are obtained from at least 3 independent experiments. Statistical significance of *P* < 0.05.

## Result

### piR-26441 was notably downregulated in OC

**Fig. 1:**
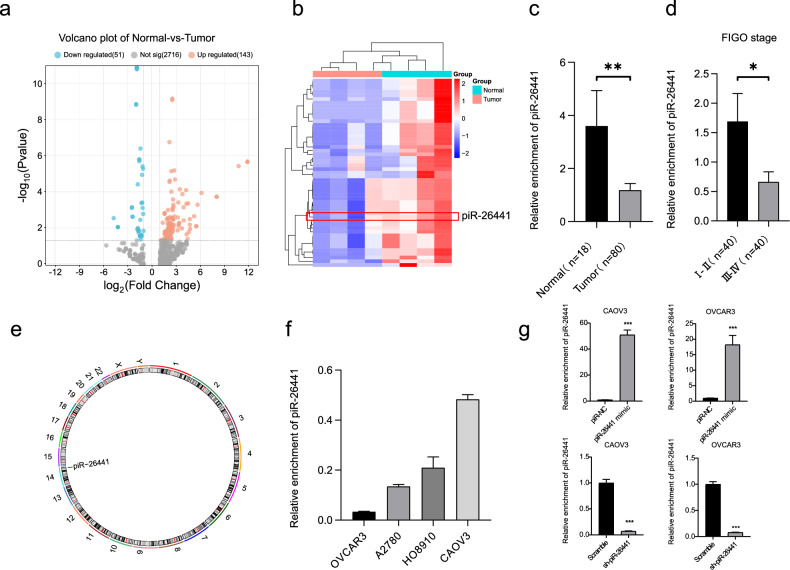
piR-26441 was notably downregulated in OC. **a** Volcano plot showing the differential expression of piRNAs between OC and normal ovarian tissue. **b** Heatmap showing down-regulated differential expression of piRNAs. **c** The expression levels of piR-26441 in OC tissue samples (*n* = 80) were lower than that in normal ovarian tissue samples (*n* = 18). **d** piR-26441 expression levels in FIGO stage III–IV diseases were lower than that in FIGO stage I-II diseases. **e** piR-26441 genomic location. **f** Expression levels of piR-26441 in OC cell lines. **g** Transfection efficiency of piR-26441 mimic and sh-piR-26441 in CAOV3 and OVCAR3. All independent experiment repeated three times. Values are presented as the mean ± SD. *p ≤ 0.05; **p ≤ 0.01; ***p ≤ 0.001.

Herein, the role of piRNAs in OC was systematically investigated. First, the expression profiles of four pairs of OC and normal ovarian tissues were analyzed using sRNA-Seq, and various piRNAs with a significant differential expression of log2 (false discovery rate) >1 in OC and normal ovarian tissues were identified (Fig. 1a). With the continued maturation of gene delivery system technology, ncRNA replacement therapy has become an emerging and promising therapeutic area [26]. Compared to ncRNA antagonism, ncRNA replacement therapy supplements molecules required for normal physiology, produces fewer side effects and restores normal biological functions [27]. Therefore, we delved into piRNAs that are lowly expressed in ovarian cancer and found piR-26441 (Fig. 1b), which is located on chromosome 14 (Fig. 1e). Compared with normal ovarian tissue, the expression levels of piR-26441 in ovarian cancer showed a low expression trend, which is consistent with the results of qRT-PCR (Fig. 1c). The results suggested that piR-26441 is downregulated in OC. Next, the relationship between piR-26441 expression and clinicopathological features of OC patients was analyzed. The down-regulation of piR-26441 expression was closely related to the OC stages, as stated by the International Federation of Gynecology and Obstetrics (FIGO). Notably, piR-26441 expression levels in FIGO stage III–IV diseases were lower than that in FIGO stage I-II diseases (Fig. 1d). Additionally, qRT-PCR results revealed that piR-26441 was minimally expressed in OVCAR3 cells and exhibited the highest expression in CAOV3 cells (Fig. 1f). Hence, mimic or shRNA was used to interfere with piR-26441 expression in CAOV3 and OVCAR3 cells, followed by an assessment of the transfection efficiency by qRT-PCR. In both CAOV3 and OVCAR3 cells, the piR-26441 mimic increased the expression of piR-26441, whereas sh-piR-26441 decreased its expression (Fig. 1g).

### piR-26441 overexpression inhibited the malignant biological behavior of OC cells

**Fig. 2:**
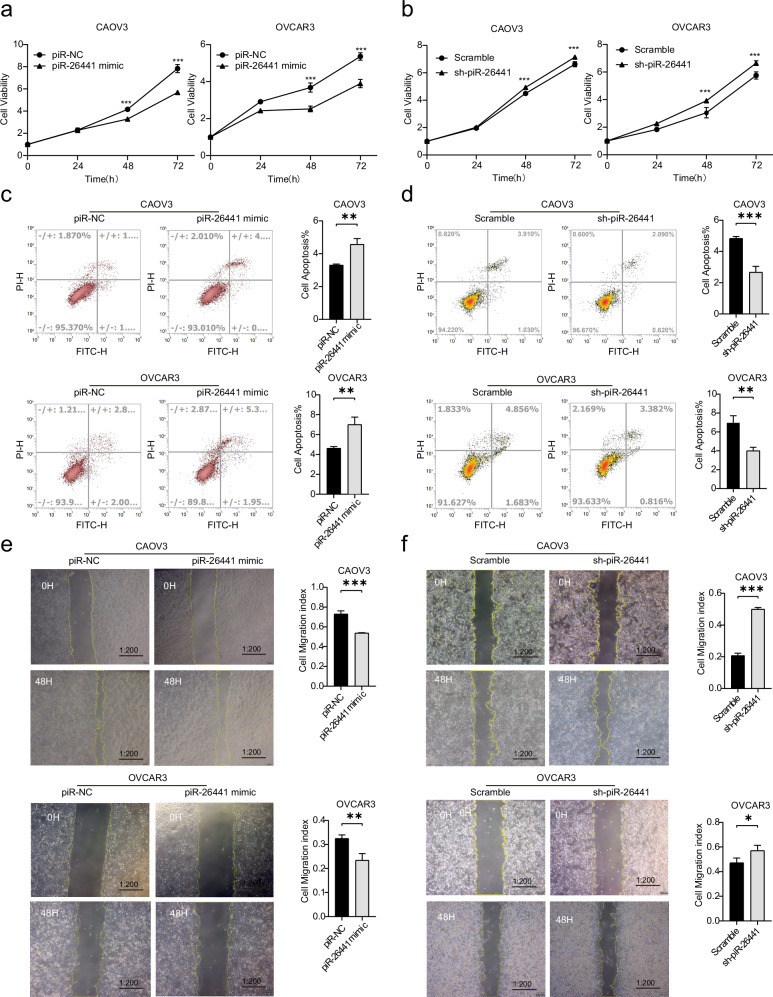
piR-26441 overexpression inhibited the malignant biological behavior of OC cells. **a** piR-26441 overexpression decreased cell viability and **e** cell migration, **c** while increasing apoptosis. **b** piR-26441 down-regulation increased cell viability and **f** cell migration, **d** while decreasing apoptosis. All independent experiment repeated three times. Values are presented as the mean ± SD. *p ≤ 0.05; **p ≤ 0.01; ***p ≤ 0.001.

piR-26441 expression in CAOV3 and OVCAR3 cells was interfered with using mimic or shRNA to analyze its biological function. CCK-8 results showed that increasing piR-26441 expression considerably decreased the proliferation rate of CAOV3 and OVCAR3 cells (Fig. 2a), whereas decreasing piR-26441 expression increased the proliferation rate of both cells (Fig. 2b). Flow cytometry analysis for CAOV3 and OVCAR3 cell apoptosis revealed that piR-26441 mimic led to an increase in the number of apoptotic cells compared to the piR-NC (Fig. 2c), and sh-piR-26441 decreased the number of apoptotic cells in comparison with the scramble (Fig. 2d). The migration ability of OC cells was examined by scratch assay. Notably, piR-26441 mimic considerably widened the trabecular surface and reduced the migration ability of CAOV3 and OVCAR3 cells in comparison to piR-NC (Fig. 2e), and sh-piR-26441 narrowed the scratches of CAOV3 and OVCAR3 cells (Fig. 2f). These results indicate that piR-26441 could inhibit the malignant biological behaviors of OC cells.

### YTHDC1 is a target of piR-26441 in OC cells

**Fig. 3:**
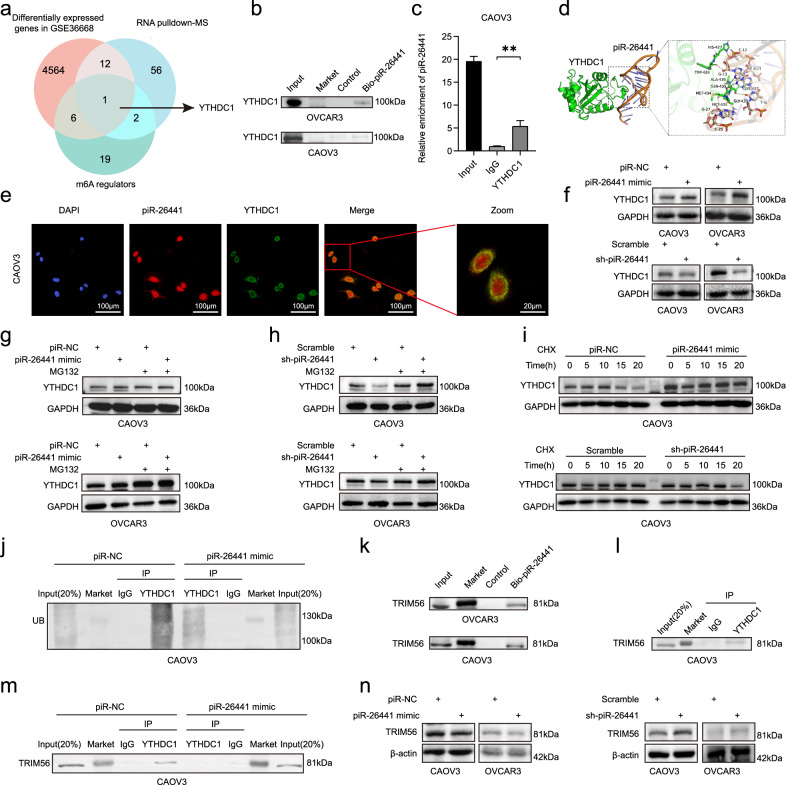
YTHDC1 is a target of piR-26441 in OC cells. **a** Overlapping analysis (Venn diagram) revealing the m6A regulators that were pulled down by biotin-labelled piR-26441 from the lysates of OVCAR3 cells in mass spectrometry (MS) assays with differentially expressed genes in OC cells (GSE36668) and m6A regulators. **b** RNA Pulldown assay showed an interaction between piR-26441 and YTHDC1. **c** RIP and qRT-PCR assays revealed an interaction between piR-26441 and YTHDC1. **d** Schematic representation of the interaction between piR-26441 and YTHDC1. **e** FISH and immunofluorescence staining showed piR-26441 (red) and YTHDC1 (green) in cultured CAOV3 cells positioning with DAPI nuclear staining (blue). **f** Western blotting showed YTHDC1 protein levels increased in CAOV3 and OVCAR3 cells after piR-26441 overexpression, which was contrary to that after knockout. **g**, **h** MG132 (20 μM) treatment reduced the regulatory effect of piR-26441 on YTHDC1 protein levels in OC cells. **i** After treatment with cycloheximide (CHX) (80 μg/ml), the degradation rate of YTHDC1 protein in piR-NC group and sh-piR-26441 group was accelerated. **j** Western blot results showed a significant reduction in the ubiquitination levels of YTHDC1 after piR-26441 overexpression. **k** RNA Pulldown assay showed an interaction between piR-26441 and TRIM56. **l** Co-IP assays revealed an interaction between YTHDC1 and TRIM56. **m** Co-IP assays revealed reduced binding of YTHDC1 to TRIM56 after piR-26441 overexpression. **n** Western blot results showed that TRIM56 protein levels decreased in CAOV3 and OVCAR3 cells after piR-26441 overexpression, which was contrary to that after knockout. All independent experiment repeated three times. Values are presented as the mean ± SD. *p ≤ 0.05; **p ≤ 0.01; ***p ≤ 0.001.

Next, the molecular mechanisms underlying piR-26441-mediated regulation of OC were investigated. The results of the RNA pulldown experiments using biotinylated piR-26441 in OVCAR3 cells showed differences in the bands of biotinylated piR-26441 pulldown compared with that of the control (Supplementary Fig. 1a), then analyzed by mass spectrometry (MS) to identify piR-26441-specific-binding proteins. MS analysis identified 71 piR-26441 pulldown proteins, which were overlaid with differentially expressed genes in GSE36668 and m6A regulators, revealing that the key gene aberrantly expressed in OCs that could bind to piR-26441 is YTHDC1 (Fig. 3a). RNA pulldown experiments in CAOV3 and OVCAR3 cells revealed that piR-26441 is specifically bound to YTHDC1 (Fig. 3b). Additionally, RIP results verified the interaction of piR-26441 with YTHDC1 in OC cells (Fig. 3c). FISH assay confirmed the nuclear co-localization of piR-26441 and YTHDC1 in CAOV3 cells (Fig. 3e). These results indicate the interaction of piR-26441 with YTHDC1 (Fig. 3d).

### piR-26441 increased YTHDC1 protein levels by reducing TRIM56-mediated ubiquitination of YTHDC1

Interestingly, in CAOV3 and OVCAR3 cells, piR-26441 mimic increased the level of the YTHDC1 protein, but not that of the mRNA, compared with those of the control. Conversely, piR-26441 knockdown decreased the YTHDC1 protein levels, without affecting its mRNA levels in OC cells (Fig. 3f, Supplementary Fig. 1b). To determine if this process is mediated by activation of proteasomal degradation, we hypothesized that piR-26441 may regulate the protein stability of YTHDC1 in OC cells. Consequently, CAOV3 and OVCAR3 cells were treated with the 26S proteasome inhibitor (MG132) following the knockdown or overexpression of piR-26441. Notably, MG132 treatment attenuated the regulatory effect of piR-26441 on YTHDC1 protein levels in OC cells (Fig. 3g, h). Furthermore, piR-26441 overexpression was prolonged, and piR-26441 silencing shortened YTHDC1 half-life in CAOV3 cells (Fig. 3i). Additionally, the levels of polyubiquitination of YTHDC1 in CAOV3 cells was reduced due to the piR-26441 mimic (Fig. 3j). Altogether, these results are evidence of that the stability of the YTHDC1 protein in OC cells was regulated by the piR-26441 gene.

MS results revealed that piR-26441 could bind specifically to the E3 ligase TRIM56 (Fig. 3k) and that piR-26441 mimic reduced the protein levels of TRIM56. Furthermore, piR-26441 knockdown upregulated TRIM56 levels (Fig. 3n). The results of co-IP showed that TRIM56 directly interacted with YTHDC1 (Fig. 3l). Notably, piR-26441 mimic attenuated the YTHDC1-TRIM56 binding (Fig. 3m). Therefore, we hypothesized that TRIM56 may be a crucial factor for piR-26441-induced YTHDC1 regulation in OC cells.

### TSFM was identified as a cascade effector of the piR-26441–YTHDC1 cascade

**Fig. 4:**
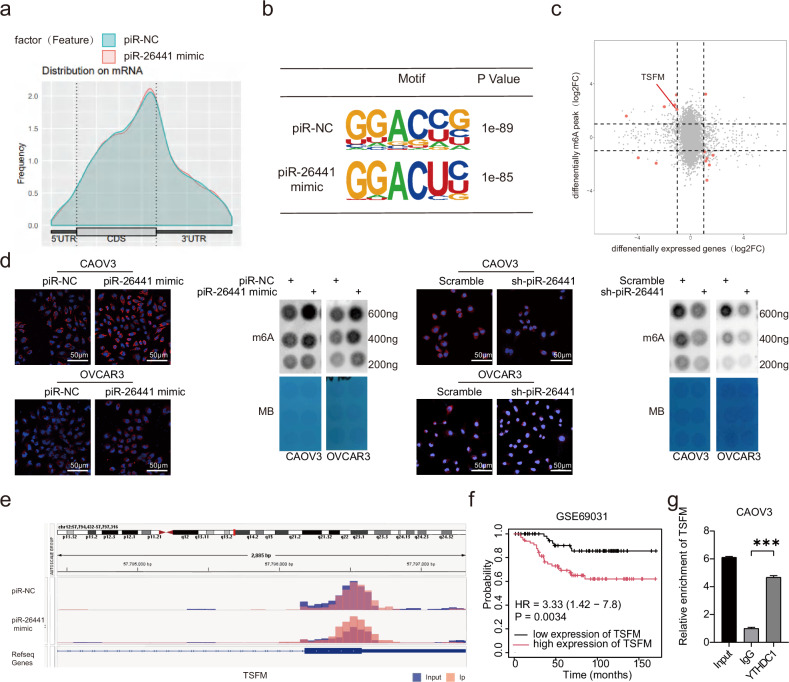
TSFM was identified as a cascade effector of the piR-26441-YTHDC1 cascade reaction. **a** The density distribution of total m6A peaks in the piR-NC and piR-26441 mimic cells. **b** Predominant consensus motif GGAC was detected in both piR-NC and piR-26441 mimic cells. **c** Volcanic figure shows the piR-NC and piR-26441 mimic differentiated m6A peaks and mRNA fold change. **d** The immunofluorescence and dot blot of m6A showed m6A levels increased in CAOV3 and OVCAR3 cells after piR-26441 overexpression, which was contrary to that after knockout. **e** Integrative Genomics Viewer (IGV) tracks display m6A abundance in TSFM transcripts in piR-NC and piR-26441 mimic cells. **f** TSFM is associated with poor prognosis of ovarian cancer. **g** RIP and qRT-PCR assays revealed an interaction between YTHDC1 and TSFM. All independent experiment repeated three times. Values are presented as the mean ± SD. *p ≤ 0.05; **p ≤ 0.01; ***p ≤ 0.001.

To further validate the effect of piR-26441 and YTHDC1 interaction on OC cells, m6A sequencing (m6A-Seq) was performed on CAOV3 cells transfected with piR-NC and piR-26441 mimic. The m6A-seq data showed that the m6A peak was distributed in coding regions (CDS) (Fig. 4a), which was consistent with previous findings [28]. As shown, GGAC was abundantly expressed at the m6A locus in both piR-NC and piR-26441 mimic cells (Fig. 4b). Notably, 14 transcripts were screened after differential m6A modification and differential gene expression cross-linking in control and piR-26441 mimic cells (Fig. 4c). The overall m6A levels were increased in piR-26441 mimic-transfected cells in comparison with that of control cells, whereas those were reduced in piR-26441-knockdown cells; this is consistent with the results of m6A immunofluorescence and dot blot experiments (Fig. 4d). Interestingly, we found that the mRNA levels of TSFM was reduced and its m6A peak was increased in piR-26441 mimic cells (Fig. 4e). High expression of TSFM is associated with poor prognosis of ovarian cancer (Fig. 4f). The RIP experiments verified the interaction of TSFM mRNA with YTHDC1 in OC cells (Fig. 4g). It was hypothesized that TSFM is likely an effector of the piR-26441–YTHDC1 cascade.

**Fig. 5:**
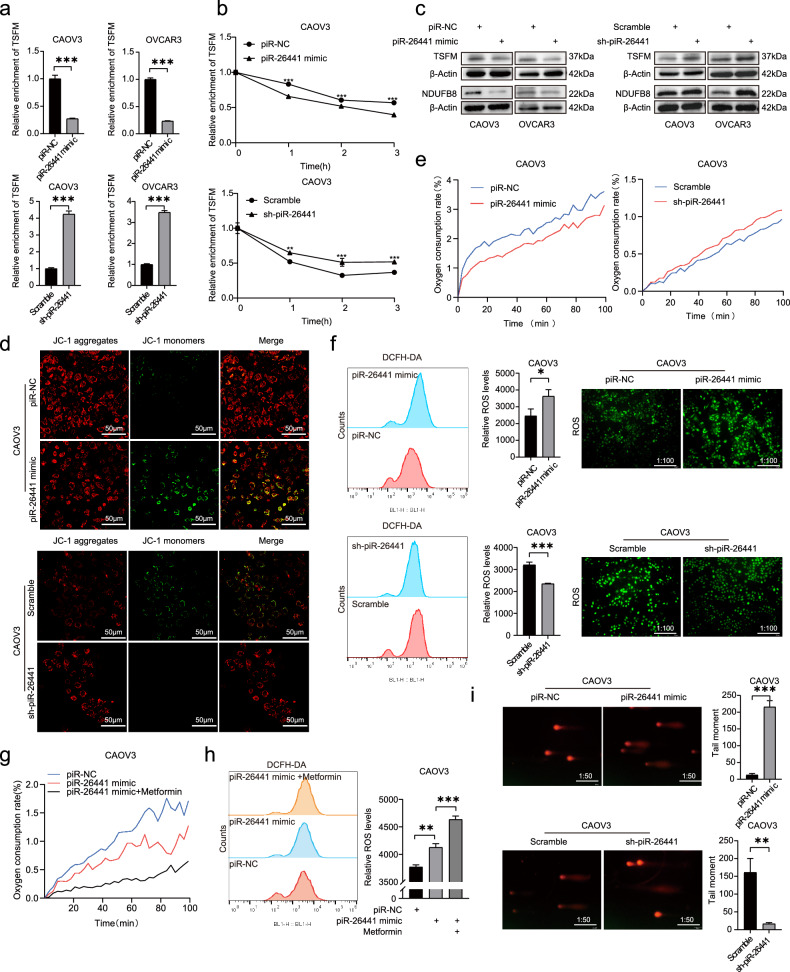
piR-26441 inhibits OXPHOS in OC cells and activates ROS to initiate the apoptotic cascade. **a** qRT-PCR results showed the RNA levels of TSFM decreased in CAOV3 and OVCAR3 cells after piR-26441 overexpression, which was contrary to that after knockout. **b** After treatment with actinomycin D (5 μg/ ml) for 0, 1, 2, and 3 h, RT-qPCR assay showed overexpression of piR-26441 shortened the mRNA half-life of TSFM in CAOV3 cells, which was contrary to that after knockout. **c** Western blot results showed TSFM and NDUFB8 protein levels decreased in CAOV3 and OVCAR3 cells after overexpression of piR-26441, which was contrary to that after knockout. **d** JC-1 staining showed that overexpression of piR-26441 decreased the mitochondrial membrane potential of CAOV3, while knockdown did the opposite. **e** The results of R01 fluorescent probe showed the oxygen consumption rate decreased in CAOV3 cells after piR-26441 overexpression, which was contrary to that after knockout. **f** Flow cytometry analysis and fluorescence microscopy results showed ROS levels increased in CAOV3 cells after piR-26441 overexpression, which was contrary to that after knockout. **g** R01 fluorescent probe results showed that overexpression of piR-26441 coupled with metformin significantly decreased the oxygen consumption rate in CAOV3 cells. **h** Flow cytometry analysis showed that overexpression of piR-26441 coupled with metformin significantly increased ROS levels in CAOV3 cells. **i** Comet assay showed DNA damage levels increased in CAOV3 cells after piR-26441 overexpression, which was contrary to that after knockout. All independent experiment repeated three times. Values are presented as the mean ± SD. *p ≤ 0.05; **p ≤ 0.01; ***p ≤ 0.001.

The m6A-Seq data showed that TSFM mRNA expression was downregulated after piR-26441 overexpression. qRT-PCR results confirmed that piR-26441 mimic downregulated TSFM expression, whereas sh-piR-26441 upregulated TSFM expression, compared with that in the control group in CAOV3 and OVCAR3 cells (Fig. 5a). Considering that YTHDC1 regulates the target gene expression by affecting mRNA stability, we used ActD to inhibit new mRNA synthesis in OC cells. The qRT-PCR results revealed that piR-26441 overexpression decreased the half-life of TSFM in CAOV3 cells, while knockdown did the opposite (Fig. 5b). Further examination of the protein expression of TSFM after piR-26441 overexpression/knockdown in CAOV3 and OVCAR3 cells by western blotting showed that piR-26441 overexpression downregulated and piR-26441 knockdown upregulated TSFM expression (Fig. 5c). These results indicate that piR-26441 reduces the stability of TSFM mRNA through YTHDC1, thereby downregulating TSFM protein expression.

### piR-26441 inhibits OXPHOS in OC cells and activates ROS to initiate the apoptotic cascade

Previous studies have reported that patients with TSFM deficiency present reduced levels of OXPHOS complexes I and IV, leading to mitochondrial dysfunction [29, 30]. Considering that piR-26441 downregulated TSFM expression through YTHDC1, it was hypothesized that piR-26441 mimic would cause mitochondrial dysfunction, thereby inhibiting mitochondrial respiration. Western blotting revealed that piR-26441 overexpression downregulated NDUFB8 expression, whereas its knockdown upregulated NDUFB8 expression (Fig. 5c). Mitochondrial membrane potential was assessed by JC-1 staining, and overexpression of piR-26441 decreased the mitochondrial membrane potential of CAOV3, while knockdown did the opposite (Fig. 5d). The cellular oxygen consumption rate was determined using the BBoxiProbe^®^ OCR Assay Kit. piR-26441 mimic markedly decreased the oxygen consumption rate of CAOV3 cells, whereas piR-26441 knockdown increased it in CAOV3 cells (Fig. 5e). We next tested the levels of glycolysis, tricarboxylic acid cycle (TCA) cycle and lipid content. The results showed that overexpression of piR-26441 decreased the extracellular acidification rate and the levels of IDH activity in CAOV3 cells, with down-regulation being the opposite, whereas there was no change in the lipid content of CAOV3 cells after overexpression or down-regulation of piR-26441 (Supplementary Fig. 2a, b, c).

Mitochondrial dysfunction is a well-known source of ROS [31]. In line with this, the results of this study showed that piR-26441 overexpression considerably increased cellular ROS levels in CAOV3 cells, and its knockdown exhibited the opposite effect (Fig. 5f). Notably, piR-26441 is synergistic with the mitochondrial respiratory chain inhibitor metformin. piR-26441 combined with metformin significantly decreased oxygen consumption rate and increased ROS levels in CAOV3 cells, comparing with piR-26441 mimic alone (Fig. 5g, h). Excessive ROS levels can lead to DNA damage and apoptosis (Fig. 5i, Fig. 2c, d). These results suggest that piR-26441 leads to mitochondrial dysfunction, which inhibits mitochondrial respiration and activates ROS in OC cells, and excessive ROS levels further lead to DNA damage and initiate apoptosis.

### piR-26441 inhibits OC cell growth through the YTHDC1/TSFM signaling axis

**Fig. 6:**
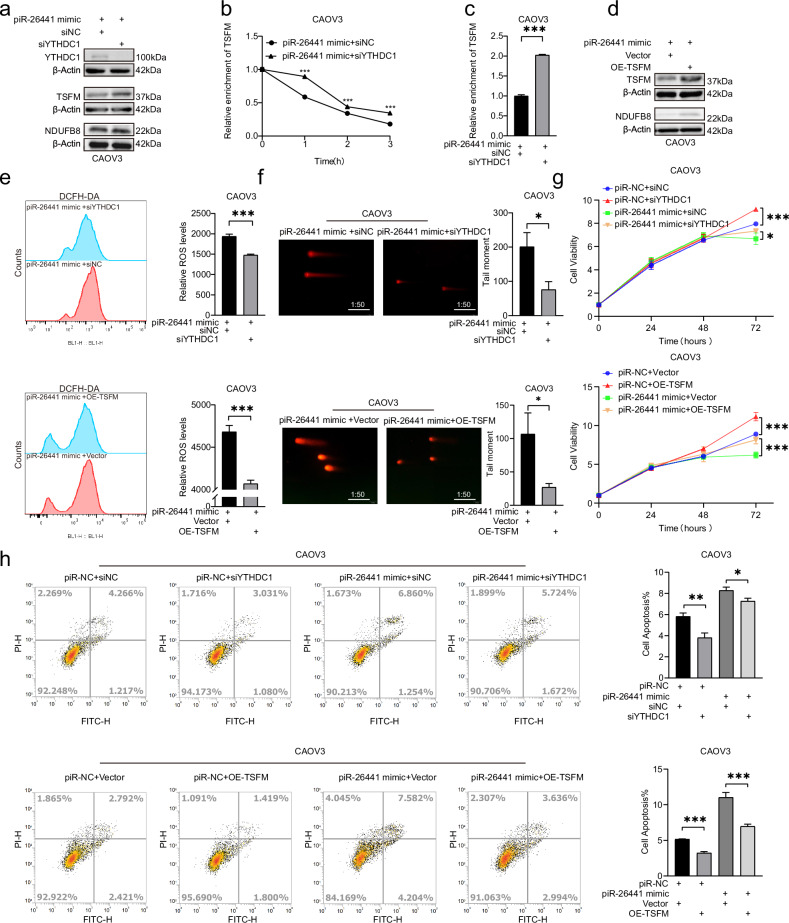
piR-26441 inhibits OC cell growth through the YTHDC1/TSFM signaling axis. **a** Western blotting showed the protein levels of YTHDC1 decreased, and the protein levels of TSFM and NDUFB8 increased in piR-26441-overexpressing cells after YTHDC1 knockdown. **b** After treatment with actinomycin D (5 μg/ml) for 0, 1, 2, and 3 h, RT-qPCR assay showed the mRNA half-life of TSFM was prolonged in piR-26441-overexpressing cells after YTHDC1 knockdown. **c** qRT-PCR results showed the RNA levels of TSFM increased in piR-26441-overexpressing cells after YTHDC1 knockdown. **d** Western blotting showed TSFM and NDUFB8 protein levels increased in piR-26441-overexpressing cells after TSFM overexpression. **e** Flow cytometry analysis results showed ROS levels decreased in piR-26441-overexpressing cells after YTHDC1 knockdown and TSFM overexpression. **f** Comet assay showed DNA damage levels decreased in piR-26441-overexpressing cells after YTHDC1 knockdown and TSFM overexpression. YTHDC1 knockdown and TSFM overexpression **g** increased cell viability and **h** decreased apoptosis of piR-26441-overexpressing cells. All independent experiment repeated three times. Values are presented as the mean ± SD. *p ≤ 0.05; **p ≤ 0.01; ***p ≤ 0.001.

The effects of piR-26441 in OC cells were determined by analyzing the YTHDC1/TSFM signaling axis. In CAOV3 cells, piR-26441 mimic increased the YTHDC1 protein levels and decreased the mRNA stability of TSFM, resulting in a decrease in both mRNA and protein levels of TSFM. In addition, the expression levels of NDUFB8 protein also showed a decrease. However, this series of changes was prevented when YTHDC1 expression was knocked down (Fig. 6a, b, c). TSFM overexpression eliminated the piR-26441 mimic-induced decrease in the TSFM and NDUFB8 protein levels (Fig. 6d). Additionally, YTHDC1 disruption and TSFM overexpression eliminated the piR-26441 overexpression-induced increased ROS levels and DNA damage in CAOV3 cells (Fig. 6e, f). Notably, CCK-8 and apoptosis assays showed that piR-26441 overexpression inhibited the growth ability and promoted apoptosis in CAOV3 and OVCAR3 cells, whereas these effects were attenuated by YTHDC1 knockdown and TSFM overexpression (Fig. 6g, h). Altogether, these results suggest that piR-26441 inhibits OC cell growth through the piR-26441/YTHDC1/TSFM axis.

### piR-26441 overexpression inhibits OC growth in vivo and in patient-derived organoids (PDOs)

**Fig. 7:**
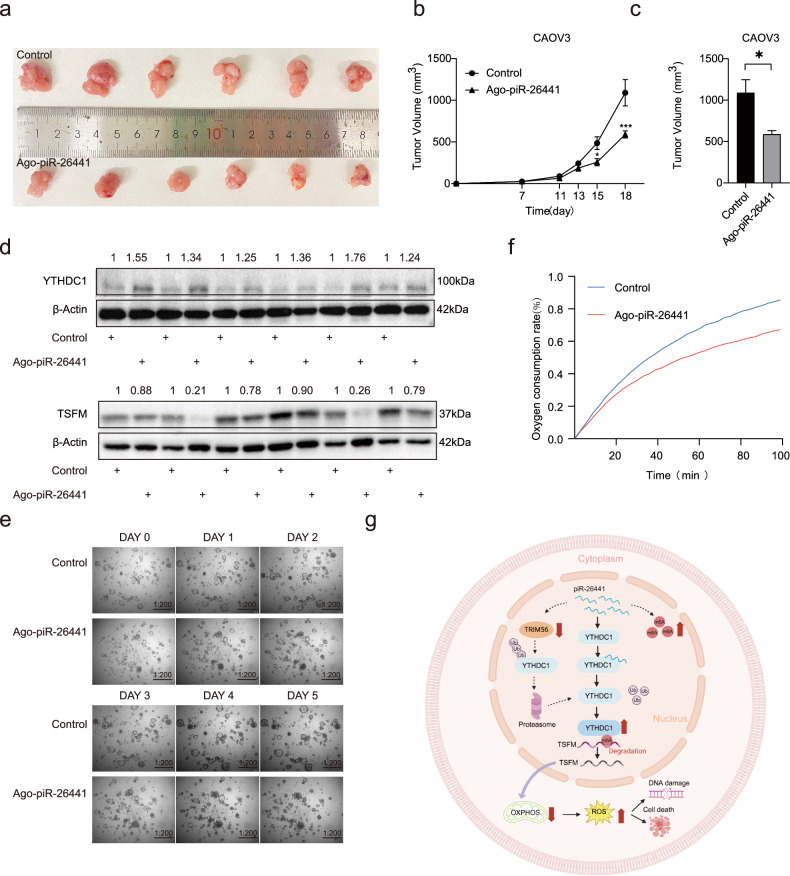
piR-26441 overexpression inhibits OC growth in vivo and in patient-derived organoids (PDOs). **a**–**c** Tumor volume of nude mice inoculated with ago-piR-26441 CAOV3 cells decreased. **d** The levels of YTHDC1 protein increased and the levels of TSFM protein decreased in subcutaneous xenografts of nude mice. **e** OC organoids became smaller in size after inoculation with ago-piR-26441. **f** The results of the R01 fluorescent probe assay showed the oxygen consumption rate decreased in the inoculated ago-piR-26441 PDOs. **g** The molecular mechanism of piR-26441. All independent experiment repeated three times. Values are presented as the mean ± SD. *p ≤ 0.05; **p ≤ 0.01; ***p ≤ 0.001.

The role of piR-26441 in the progression of OC was investigated using the mouse xenograft model. Notably, mice injected with CAOV3 cells and ago-piR-26441 exhibited a slower rate of tumor formation and smaller tumor size compared with those of the control (Fig. 7a–c). In ago-piR-26441-injected xenografts, YTHDC1 protein levels increased, and those of TSFM decreased (Fig. 7d). Herein, the OC organoid model was established, which better maintains the morphology and biological characteristics of ovarian tumor cells. The OC tissues were digested into 3–10 cell clusters and seeded into a three-dimensional medium followed by ago-piR-26441 addition. The ago-piR-26441 group presented smaller organoid diameters than that in the control group and ago-piR-26441 markedly decreased the oxygen consumption rate of PDO (Fig. 7e, f). These results indicate that piR-26441 overexpression inhibited the growth of OC.

## Discussion

Among the three major gynecological malignancies, OC presents a mortality rate of up to 70% despite chemotherapy and targeted therapy [32]. In the past decade, the novel theory of “cellular energy dysregulation” was proposed, which focused on the metabolic support for cancer cell growth, proliferation, invasion, and metastasis, and has become one of the biological targets for developing novel cancer therapies [33]. Although previous studies report the Warburg effect to be the dominant metabolic mode of tumor cells [13], current evidence contradicts this notion, suggesting that not every tumor exhibits this effect [34, 35]. The metabolism of OC has been reported to exhibit heterogeneity, indicating the use of another metabolic pathway for energy generation, such as OXPHOS [35, 36]. Burdett et al. reported that upregulation of OXPHOS and glucose metabolism pathways in primary or recurrent OC autopsy samples positively correlated with advanced high-grade serous carcinoma (HGSC) and post-chemotherapy homologous recombination (HR) defects. Furthermore, the proteomic analysis showed that advanced HGSC and HR-deficient tumors relied on OXPHOS to meet cellular energy demands and can be used as potential predictors of poly adenosine diphosphate ribose polymerase inhibitors (PARPi) response [37]. The metabolic reprogramming process is considered a promising therapeutic target, and the findings of this study provide a potential approach for OC treatment from a metabolic perspective.

Recently, many studies have highlighted the crucial roles of piRNAs in cancer, where they regulate gene expression mainly through TGS, PTGS, and protein interaction. Aberrant expression of piRNAs serves as a key regulator of cancer cell proliferation, apoptosis, invasion, and migration, suggesting that it may be a potential therapeutic target for OC. For example, piR-52207 has been reported to promote cell proliferation, migration, and tumorigenesis in endometrioid OC cells mainly by targeting (nucleoside diphosphate linked moiety X)-type motif 4, 5-methyltetrahydrofolate-homocysteine methyltransferase, eukaryotic translation initiation factor 2 subunit gamma, and M-phase phosphoprotein 8. Reportedly, piR-33733 and piR-52207 have been found to promote cell proliferation, migration, and tumorigenesis in plasma ovarian carcinoma. piR-33733 targets lipoic acid synthetase, while piR-52207 binds to actin-related protein 10 and Pleckstrin homology domain-containing A5. These interactions lead to an increase in anti-apoptotic and a decrease in pro-apoptotic proteins, thereby contributing to OC development [38]. In this study, the determination of the specific function and potential mechanism of piR-26441 in OC revealed that piR-26441 destabilized the TSFM mRNA by interacting with YTHDC1, leading to mitochondrial dysfunction, inhibition of mitochondrial respiration, and ultimately, inhibition of OC development.

YTHDC1 is a member of the YTH structural domain-containing protein family, which plays a crucial role in the mRNA methylation mechanism. YTHDC1 is a “reader” of m6A methyltransferases and is associated with functions including mRNA splicing, nuclear export, and stability, along with playing a role in anti-cancer. In OC, YTHDC1 increases the stability of phosphoinositide-3-kinase regulatory subunit 1 in an m6A-dependent manner, and subsequently inhibits glucosidase II alpha subunit expression in N-glycan biosynthesis through signal transducer and activator of transcription 3 signaling, thereby suppressing the tumor growth [39]. Additionally, YTHDC1 has been reported to inhibit tumor development by regulating mitochondrial energy metabolism in tumor cells. Reportedly, YTHDC1 and glucose transporter 3/ring finger protein 183 form a feedback loop to regulate bladder cancer development and glucose metabolism [40]. Herein, the gain- and loss-of-function experiments showed that YTHDC1 inhibited mitochondrial respiration and malignant progression of OC cells, suggesting the anti-cancer role of YTHDC1 in OC.

TSFM is a mitochondrial translational elongation factor that participates in the elongation step of mitochondrial translation by binding and stabilizing the Tu translation elongation factor, mitochondrial (TUFM). Pathogenic variants in any of the four nuclear genes encoding the mitochondrial elongation factors EFG1, EFG2, TUFM, and TSFM have been implicated in causing severe mitochondrial disease [41]. In a study, biochemical analyses of mitochondrial respiratory chain enzyme activity performed on cardiac homogenates from patients with defective TSFM genes showed reduced levels of OXPHOS complexes I and IV, whereas the levels of complexes II and III were found to be normal [30]. TSFM plays an oncogenic role in OC, and according to the Kaplan–Meier survival curves, the survival rate of the high TSFM expression group has been reported to be significantly lower than that of the low TSFM expression group, with the expression of TSFM in the normal ovarian tissues being significantly lower than that in the OC tissues [42], which is consistent with the results of this study. Herein, the mRNA stability and protein expression of TSFM decreased, resulting in mitochondrial dysfunction. TSFM knockdown led to an increase in ROS levels in OC cells, and excessive ROS levels caused DNA damage and induced apoptosis.

Here, our results demonstrate that cancer inhibitor piR-26441 was downregulated in the OC tissues. High piR-26441 expression was associated with increased apoptosis in OC cells. piR-26441 interacted with YTHDC1 to promote m6A modification of TSFM, thereby destabilizing TSFM mRNA and reducing its protein levels. Consistent with this, a dynamic mitochondrial OXPHOS process in OC cells (that is, the decrease or increase of mitochondrial complex I and oxygen consumption rate of OC cells following piR-26441 overexpression or knockdown, respectively) was observed. Additionally, TSFM downregulation inhibited mitochondrial respiration and activated ROS in OC cells, and excessive ROS levels further led to DNA damage and initiated apoptosis. We also visualized the dynamic changes in mitochondrial morphology and ROS production (that is, when piR-26441 was overexpressed, JC-1 as monomers produced green fluorescence and the intensity of ROS fluorescence was increased; whereas when piR-26441 was knocked down, JC-1 as aggregates produced red fluorescence and the intensity of ROS fluorescence was diminished). We therefore speculated that piR-26441 as a potential target for developing the treatment strategy for OC, utilizing the piR-26441/YTHDC1/TSFM axis.

Tumor cells often undergo complex metabolic reprogramming during ontogeny and progression to adapt to rapid growth, escape apoptosis, and resist therapeutic stress [43]. In this study, piR-26441 not only affects mitochondrial OXPHOS via YTHDC1-TSFM in OC cells, but also affects glycolysis and TCA cycle. This provides insights for a more comprehensive understanding of the role of piR-26441 in remodelling the metabolism of tumor cells and suggests new therapeutic strategies targeting multiple metabolic weaknesses. It was shown that drugs such as phenelzine or metformin can inhibit components of the mitochondrial respiratory chain [44, 45]. In this study, it was also demonstrated that metformin and piR-26441 can synergistically affect mitochondrial function, amplifying the effects of piR-26441 overexpression. This provides valuable insight into that piR-26441 in combination with mitochondrial respiratory chain inhibitors can more effectively disrupt metabolic function in OC cells.

In contrast to synthetic compounds or ASOs, piR-26441 functions as a natural tumor suppressor in humans. Once introduced into the body, the subsequent processing of the RNA and selection of downstream targets are consistent with the mechanism observed in vivo. This theoretically minimises the possibility of side effects in humans. The findings of this study indicate that ago-piR-26441 has the potential to inhibit mitochondrial respiration and tumor growth in PDOs. PDOs represent a valuable preclinical model for drug research, offering insights into the real-world response of patients to pharmacological agents. However, pharmacology, pharmacokinetics, toxicology, long-term toxicity experiments and clinical trials are still needed before clinical application. This research is still far from clinical application.

## Supplementary information

Supplementary Figure

Original western blots

## Data Availability

The data that support the findings of this study are available from the corresponding author upon reasonable request.

## References

[1] Siegel RL, Giaquinto AN, Jemal A. Cancer statistics, 2024. CA: Cancer J. Clin. 2024;74:12–49. 38230766 10.3322/caac.21820

[2] Kim SI, Kim JW. Role of surgery and hyperthermic intraperitoneal chemotherapy in ovarian cancer. ESMO open. 2021;6:100149. 33984680 10.1016/j.esmoop.2021.100149 PMC8314869

[3] Modugno F, Edwards RP. Ovarian cancer: prevention, detection, and treatment of the disease and its recurrence. Molecular mechanisms and personalized medicine meeting report. Int. J. Gynecol. Cancer : J. Int. Gynecol. Cancer Soc. 2012;22:S45–57. 10.1097/IGC.0b013e31826bd1f2 PMC3460381 23013733

[4] Tan Q, Liu H, Xu J, Mo Y, Dai F. Integrated analysis of tumor-associated macrophage infiltration and prognosis in ovarian cancer. Aging. 2021;13:23210–32. 34633990 10.18632/aging.203613 PMC8544311

[5] Liu S, Wu M, Wang F. Research Progress in Prognostic Factors and Biomarkers of Ovarian Cancer. J. Cancer. 2021;12:3976–96. 34093804 10.7150/jca.47695 PMC8176232

[6] Liu P, Dong Y, Gu J, Puthiyakunnon S, Wu Y, Chen XG. Developmental piRNA profiles of the invasive vector mosquito Aedes albopictus. Parasites Vectors. 2016;9:524. 27686069 10.1186/s13071-016-1815-8 PMC5041409

[7] Liu Y, Dou M, Song X, Dong Y, Liu S, Liu H, et al. The emerging role of the piRNA/piwi complex in cancer. Mol. Cancer. 2019;18:123. 31399034 10.1186/s12943-019-1052-9 PMC6688334

[8] Yin J, Jiang XY, Qi W, Ji CG, Xie XL, Zhang DX, et al. piR-823 contributes to colorectal tumorigenesis by enhancing the transcriptional activity of HSF1. Cancer Sci. 2017;108:1746–56. 28618124 10.1111/cas.13300 PMC5581525

[9] Sun T, Wu R, Ming L. The role of m6A RNA methylation in cancer. Biomed. Pharmacother. 2019;112:108613. 30784918 10.1016/j.biopha.2019.108613

[10] Han H, Fan G, Song S, Jiang Y, Qian C, Zhang W, et al. piRNA-30473 contributes to tumorigenesis and poor prognosis by regulating m6A RNA methylation in DLBCL. Blood. 2021;137:1603–14. 32967010 10.1182/blood.2019003764

[11] Gentric G, Mieulet V, Mechta-Grigoriou F. Heterogeneity in Cancer Metabolism: New Concepts in an Old Field. Antioxid. Redox Signaling 2017;26:462–85. 10.1089/ars.2016.6750 PMC5359687 27228792

[12] Vander Heiden MG, DeBerardinis RJ. Understanding the Intersections between Metabolism and Cancer Biology. Cell. 2017;168:657–69. 28187287 10.1016/j.cell.2016.12.039 PMC5329766

[13] Warburg O. On the origin of cancer cells. Science 1956;123:309–14. 13298683 10.1126/science.123.3191.309

[14] Caro P, Kishan AU, Norberg E, Stanley IA, Chapuy B, Ficarro SB, et al. Metabolic signatures uncover distinct targets in molecular subsets of diffuse large B cell lymphoma. Cancer Cell. 2012;22:547–60. 23079663 10.1016/j.ccr.2012.08.014 PMC3479446

[15] Vazquez F, Lim JH, Chim H, Bhalla K, Girnun G, Pierce K, et al. PGC1α expression defines a subset of human melanoma tumors with increased mitochondrial capacity and resistance to oxidative stress. Cancer Cell. 2013;23:287–301. 23416000 10.1016/j.ccr.2012.11.020 PMC3708305

[16] Camarda R, Zhou AY, Kohnz RA, Balakrishnan S, Mahieu C, Anderton B, et al. Inhibition of fatty acid oxidation as a therapy for MYC-overexpressing triple-negative breast cancer. Nat. Med. 2016;22:427–32. 26950360 10.1038/nm.4055 PMC4892846

[17] Hensley CT, Faubert B, Yuan Q, Lev-Cohain N, Jin E, Kim J, et al. Metabolic Heterogeneity in Human Lung Tumors. Cell. 2016;164:681–94. 26853473 10.1016/j.cell.2015.12.034 PMC4752889

[18] Gentric G, Kieffer Y, Mieulet V, Goundiam O, Bonneau C, Nemati F, et al. PML-Regulated Mitochondrial Metabolism Enhances Chemosensitivity in Human Ovarian Cancers. Cell Metab. 2019;29:156–73.e10. 30244973 10.1016/j.cmet.2018.09.002 PMC6331342

[19] Huang H, Gao Q, Peng X, Choi SY, Sarma K, Ren H, et al. piRNA-associated germline nuage formation and spermatogenesis require MitoPLD profusogenic mitochondrial-surface lipid signaling. Dev. Cell. 2011;20:376–87. 21397848 10.1016/j.devcel.2011.01.004 PMC3061402

[20] Huang PJ, Liu YC, Lee CC, Lin WC, Gan RR, Lyu PC, et al. DSAP: deep-sequencing small RNA analysis pipeline. Nucleic Acids Res. 2010;38:W385–91. 20478825 10.1093/nar/gkq392 PMC2896168

[21] Li Q, Xie B, Chen X, Lu B, Chen S, Sheng X, et al. SNORD6 promotes cervical cancer progression by accelerating E6-mediated p53 degradation. Cell Death Discov. 2023;9:192. 37369687 10.1038/s41420-023-01488-w PMC10300194

[22] Dominissini D, Moshitch-Moshkovitz S, Amariglio N, Rechavi G. Transcriptome-Wide Mapping of N^6^-Methyladenosine by m^6^A-Seq. Methods Enzymol.2015;560:131–47. 26253969 10.1016/bs.mie.2015.03.001

[23] Nanki Y, Chiyoda T, Hirasawa A, Ookubo A, Itoh M, Ueno M, et al. Patient-derived ovarian cancer organoids capture the genomic profiles of primary tumours applicable for drug sensitivity and resistance testing. Sci. Rep. 2020;10:12581. 32724113 10.1038/s41598-020-69488-9 PMC7387538

[24] Senkowski W, Gall-Mas L, Falco MM, Li Y, Lavikka K, Kriegbaum MC, et al. A platform for efficient establishment and drug-response profiling of high-grade serous ovarian cancer organoids. Dev. Cell. 2023;58:1106–21.e7. 37148882 10.1016/j.devcel.2023.04.012 PMC10281085

[25] Zhang S, Iyer S, Ran H, Dolgalev I, Gu S, Wei W, et al. Genetically Defined, Syngeneic Organoid Platform for Developing Combination Therapies for Ovarian Cancer. Cancer Discov. 2021;11:362–83. 33158842 10.1158/2159-8290.CD-20-0455 PMC7858239

[26] Sun L, Liu H, Ye Y, Lei Y, Islam R, Tan S, et al. Smart nanoparticles for cancer therapy. Signal Transduct. Target. Ther. 2023;8:418. 37919282 10.1038/s41392-023-01642-x PMC10622502

[27] Winkle M, El-Daly SM, Fabbri M, Calin GA. Noncoding RNA therapeutics - challenges and potential solutions. Nat. Rev. Drug Discov. 2021;20:629–51. 34145432 10.1038/s41573-021-00219-z PMC8212082

[28] Dominissini D, Moshitch-Moshkovitz S, Schwartz S, Salmon-Divon M, Ungar L, Osenberg S, et al. Topology of the human and mouse m6A RNA methylomes revealed by m6A-seq. Nature. 2012;485:201–6. 22575960 10.1038/nature11112

[29] Smeitink JA, Elpeleg O, Antonicka H, Diepstra H, Saada A, Smits P, et al. Distinct clinical phenotypes associated with a mutation in the mitochondrial translation elongation factor EFTs. Am. J. Hum. Genet. 2006;79:869–77. 17033963 10.1086/508434 PMC1698578

[30] Perli E, Pisano A, Glasgow RIC, Carbo M, Hardy SA, Falkous G, et al. Novel compound mutations in the mitochondrial translation elongation factor (TSFM) gene cause severe cardiomyopathy with myocardial fibro-adipose replacement. Sci. Rep. 2019;9:5108. 30911037 10.1038/s41598-019-41483-9 PMC6434145

[31] Turrens JF. Mitochondrial formation of reactive oxygen species. J. Physiol. 2003;552:335–44. 14561818 10.1113/jphysiol.2003.049478 PMC2343396

[32] Nantasupha C, Thonusin C, Charoenkwan K, Chattipakorn S, Chattipakorn N. Metabolic reprogramming in epithelial ovarian cancer. Am. J. Transl. Res. 2021;13:9950–73. 34650675 PMC8507042

[33] Hanahan D, Weinberg RA. Hallmarks of cancer: the next generation. Cell. 2011;144:646–74. 21376230 10.1016/j.cell.2011.02.013

[34] Kozlovski I, Siegfried Z, Amar-Schwartz A, Karni R. The role of RNA alternative splicing in regulating cancer metabolism. Hum. Genet. 2017;136:1113–27. 28429085 10.1007/s00439-017-1803-x

[35] Suh DH, Kim HS, Kim B, Song YS. Metabolic orchestration between cancer cells and tumor microenvironment as a co-evolutionary source of chemoresistance in ovarian cancer: a therapeutic implication. Biochem. Pharmacol. 2014;92:43–54. 25168677 10.1016/j.bcp.2014.08.011

[36] Nayak AP, Kapur A, Barroilhet L, Patankar MS. Oxidative Phosphorylation: A Target for Novel Therapeutic Strategies Against Ovarian Cancer. Cancers. 2018;10:337. 30231564 10.3390/cancers10090337 PMC6162441

[37] Burdett NL, Willis MO, Alsop K, Hunt AL, Pandey A, Hamilton PT, et al. Multiomic analysis of homologous recombination-deficient end-stage high-grade serous ovarian cancer. Nat. Genet. 2023;55:437–50. 36849657 10.1038/s41588-023-01320-2

[38] Singh G, Roy J, Rout P, Mallick B. Genome-wide profiling of the PIWI-interacting RNA-mRNA regulatory networks in epithelial ovarian cancers. PloS one. 2018;13:e0190485. 29320577 10.1371/journal.pone.0190485 PMC5761873

[39] Wang X, Chen Q, Bing Z, Zhou S, Xu Z, Hou Y, et al. Low expression of m6A reader YTHDC1 promotes progression of ovarian cancer via PIK3R1/STAT3/GANAB axis. Int. J. Biol. Sci. 2023;19:4672–88. 37781028 10.7150/ijbs.81595 PMC10535707

[40] Yan B, Li X, Peng M, Zuo Y, Wang Y, Liu P, et al. The YTHDC1/GLUT3/RNF183 axis forms a positive feedback loop that modulates glucose metabolism and bladder cancer progression. Exp. Mol. Med. 2023;55:1145–58. 37258572 10.1038/s12276-023-00997-z PMC10318083

[41] Boczonadi V, Horvath R. Mitochondria: impaired mitochondrial translation in human disease. Int. J. Biochem. Cell Biol. 2014;48:77–84. 24412566 10.1016/j.biocel.2013.12.011 PMC3988845

[42] Xiang J, Su R, Wu S, Zhou L. Construction of a prognostic signature for serous ovarian cancer based on lactate metabolism-related genes. Front. Oncol. 2022;12:967342. 36185201 10.3389/fonc.2022.967342 PMC9520471

[43] Ohshima K, Morii E Metabolic Reprogramming of Cancer Cells during Tumor Progression and Metastasis. Metabolites. 2021;11. 10.3390/metabo11010028 PMC7824065 33401771

[44] Singh IN, Gilmer LK, Miller DM, Cebak JE, Wang JA, Hall ED. Phenelzine mitochondrial functional preservation and neuroprotection after traumatic brain injury related to scavenging of the lipid peroxidation-derived aldehyde 4-hydroxy-2-nonenal. J. Cereb. Blood Flow. Metab. : Off. J. Int. Soc. Cereb. Blood Flow. Metab. 2013;33:593–9. 10.1038/jcbfm.2012.211 PMC3618398 23321786

[45] Feng J, Wang X, Ye X, Ares I, Lopez-Torres B, Martínez M, et al. Mitochondria as an important target of metformin: The mechanism of action, toxic and side effects, and new therapeutic applications. Pharmacol. Res. 2022;177:106114. 35124206 10.1016/j.phrs.2022.106114

