# Supplementary material for: piR-26441 inhibits mitochondrial oxidative phosphorylation and tumorigenesis in ovarian cancer through m6A modification by interacting with YTHDC1
Source: Cell Death Dis. 2025 Jan 18;16(1):25. doi: 10.1038/s41419-025-07340-6 (PMC11742951; doi:10.1038/s41419-025-07340-6)
Supplement: Supplementary file 1 — Supplementary Figure [file 41419_2025_7340_MOESM1_ESM.docx]

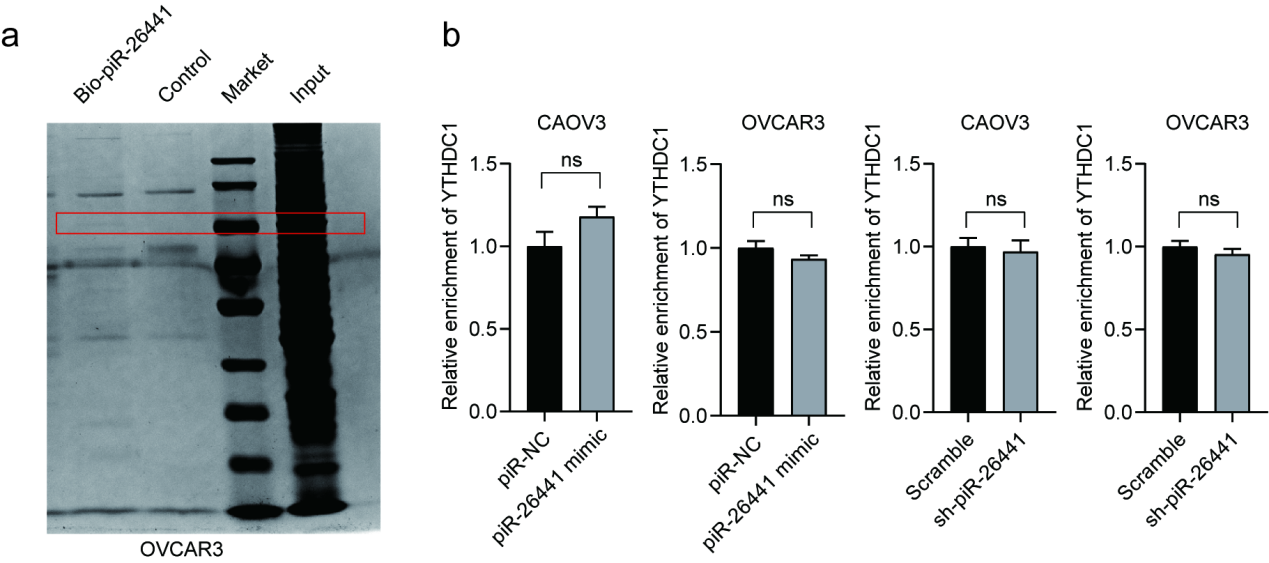


**Supplementary Figure 1 a** RNA pulldown results showed differences in the bands of biotinylated piR-26441 pulldown compared with that of the control. **b** qRT-PCR results showed that the RNA levels of YTHDC1 in CAOV3 and OVCAR3 cells did not change after overexpression or knockout of piR-26441.


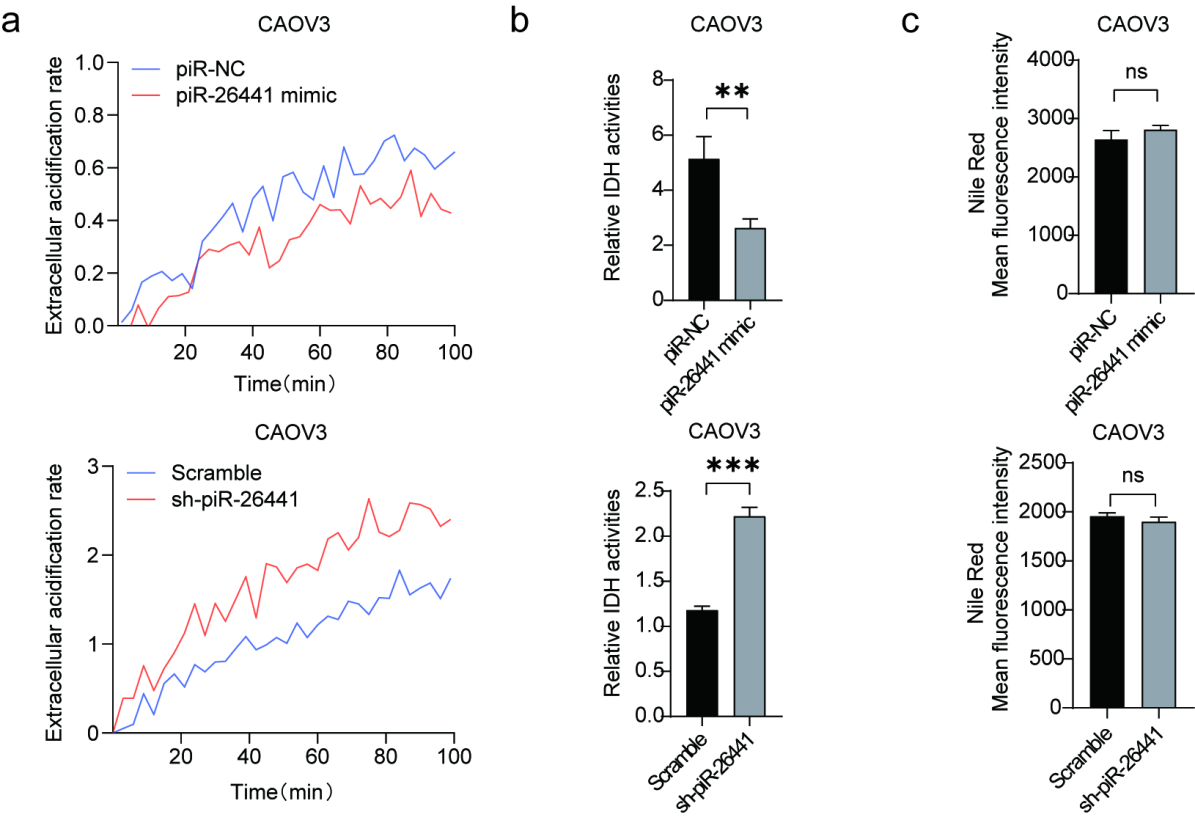


**Supplementary Figure 2 a** Glycolysis levels were assayed using the Extracellular acidification Rate Assay Kit, which showed that overexpression of piR-26441 decreased the rate of extracellular acidification in CAOV3 cells, whereas knockdown of piR-26441 did the opposite. **b** Tricarboxylic acid cycle levels were assayed using a mitochondrial isocitrate dehydrogenase (ICDHm) activity assay kit, which showed that overexpression of piR-26441 resulted in a decrease in IDH activity in CAOV3 cells, and knockdown was the opposite. **c** The lipid content of the cells was detected by Nile Red staining, and flow cytometry analysis showed that the lipid content of CAOV3 cells did not change after overexpression or knockdown of piR-26441.
