# Supplementary material for: piR-26441 inhibits mitochondrial oxidative phosphorylation and tumorigenesis in ovarian cancer through m6A modification by interacting with YTHDC1
Source: Cell Death Dis. 2025 Jan 18;16(1):25. doi: 10.1038/s41419-025-07340-6 (PMC11742951; doi:10.1038/s41419-025-07340-6)
Supplement: Supplementary file 2 — Original western blots [file 41419_2025_7340_MOESM2_ESM.pptx]

## Slide 1
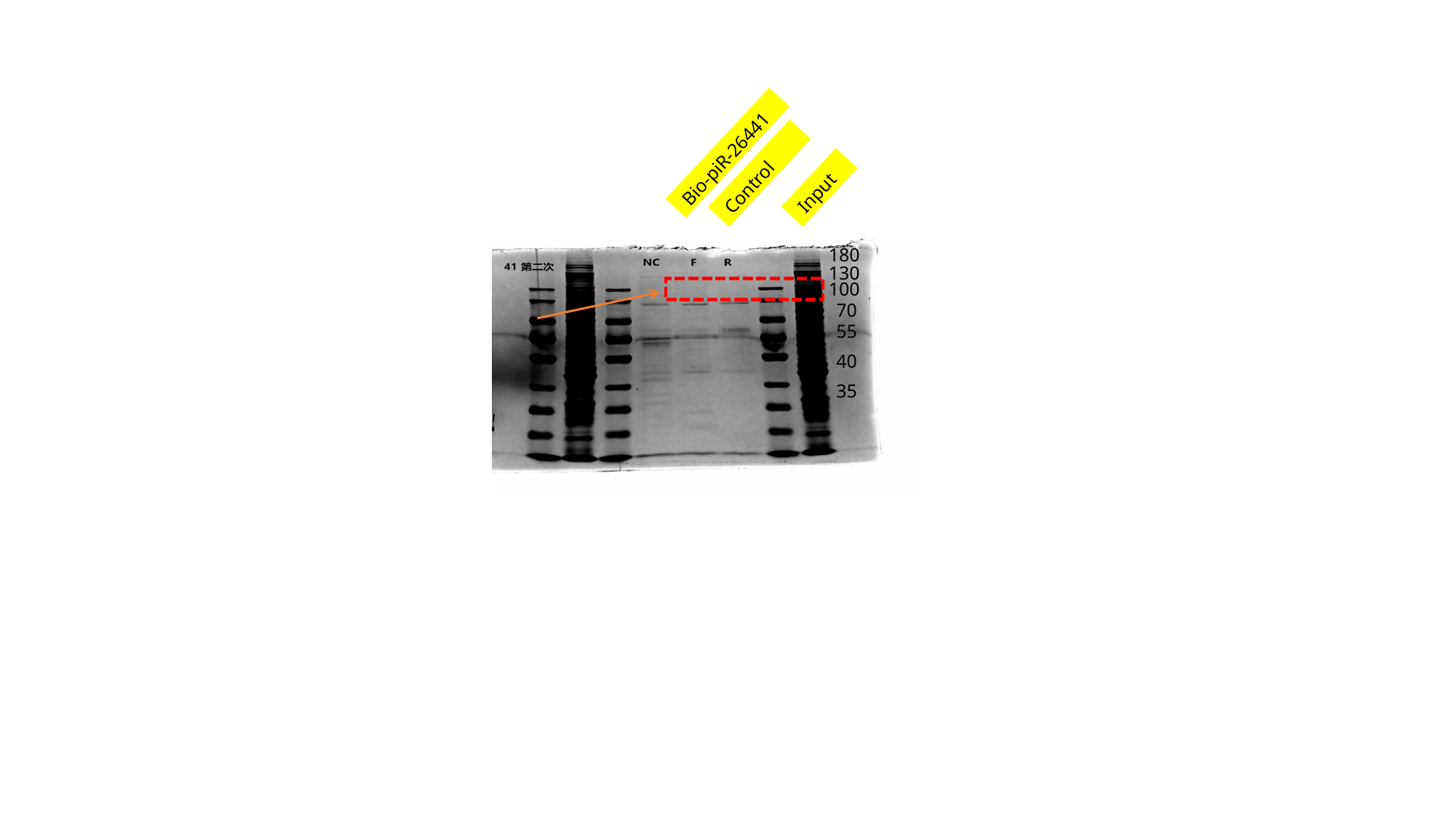

Bio-piR-26441
Control
Input
180
130
100
70
55
40
35

## Slide 2
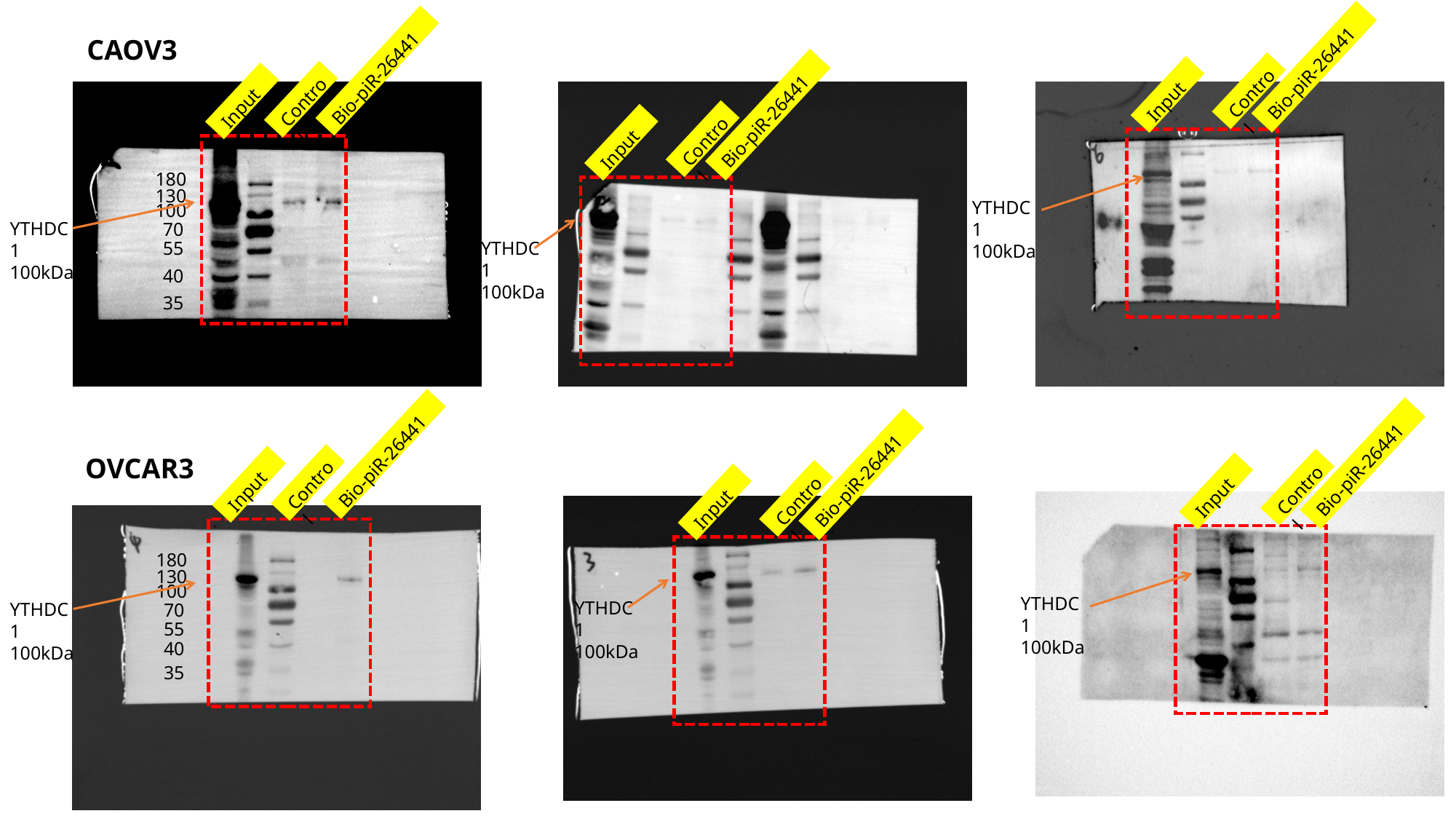

Bio-piR-26441
Control
Input
YTHDC1 100kDa
Bio-piR-26441
Control
Input
180
130
100
70
55
40
35
YTHDC1 100kDa
CAOV3
Bio-piR-26441
Control
Input
YTHDC1 100kDa
Bio-piR-26441
Control
Input
180
130
100
70
55
40
35
YTHDC1 100kDa
Bio-piR-26441
Control
Input
YTHDC1 100kDa
Bio-piR-26441
Control
Input
YTHDC1 100kDa
OVCAR3

## Slide 3
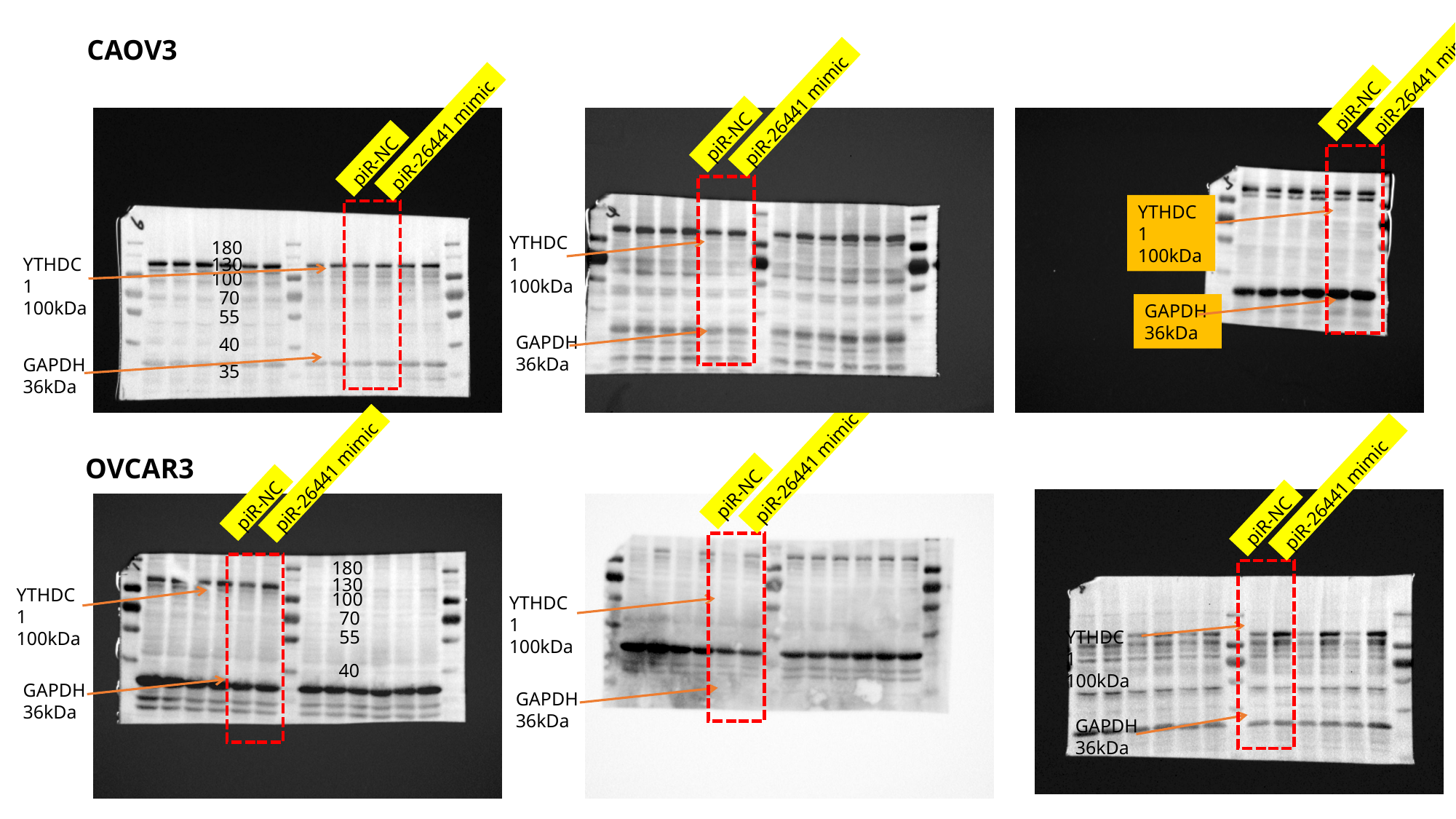

piR-26441 mimic
piR-NC
YTHDC1 100kDa
GAPDH 36kDa
piR-26441 mimic
piR-NC
YTHDC1 100kDa
GAPDH 36kDa
CAOV3
piR-26441 mimic
piR-NC
YTHDC1 100kDa
GAPDH 36kDa
180
130
100
70
55
40
35
piR-26441 mimic
piR-NC
YTHDC1 100kDa
GAPDH 36kDa
piR-26441 mimic
piR-NC
180
130
100
70
55
40
YTHDC1 100kDa
GAPDH 36kDa
piR-26441 mimic
piR-NC
YTHDC1 100kDa
GAPDH 36kDa
OVCAR3

## Slide 4
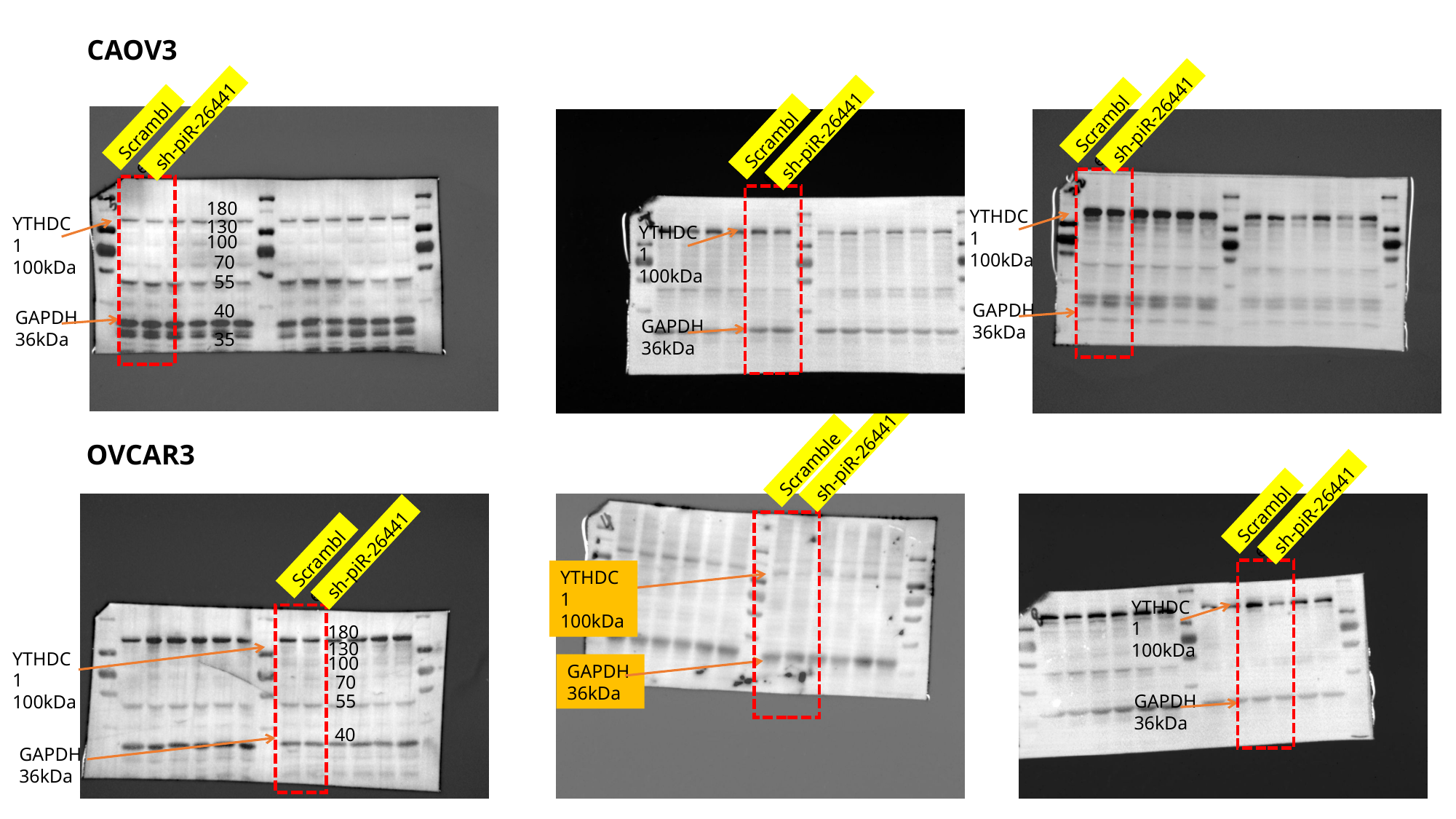

CAOV3
sh-piR-26441
Scramble
YTHDC1 100kDa
GAPDH 36kDa
sh-piR-26441
Scramble
YTHDC1 100kDa
GAPDH 36kDa
180
130
100
70
55
40
35
sh-piR-26441
Scramble
YTHDC1 100kDa
GAPDH 36kDa
sh-piR-26441
Scramble
YTHDC1 100kDa
GAPDH 36kDa
OVCAR3
sh-piR-26441
Scramble
YTHDC1 100kDa
GAPDH 36kDa
sh-piR-26441
Scramble
180
130
100
70
55
40
YTHDC1 100kDa
GAPDH 36kDa

## Slide 5
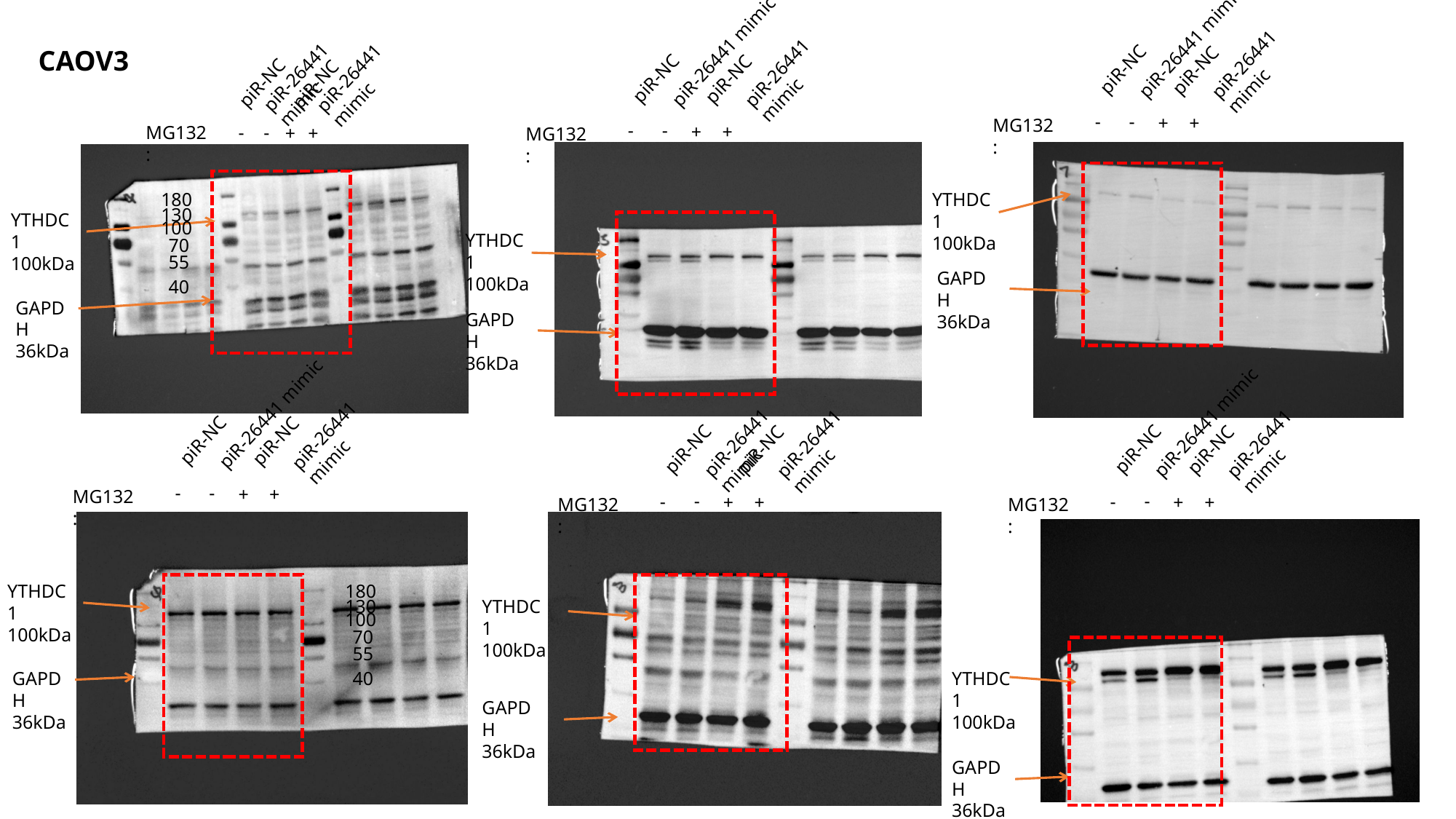

piR-26441 mimic
piR-26441 mimic
piR-NC
piR-NC
-
-
+
+
MG132:
YTHDC1 100kDa
GAPDH 36kDa
piR-26441 mimic
piR-26441 mimic
piR-NC
piR-NC
-
-
+
+
MG132:
YTHDC1 100kDa
GAPDH 36kDa
piR-26441 mimic
piR-26441 mimic
piR-NC
piR-NC
-
-
+
+
YTHDC1 100kDa
GAPDH 36kDa
MG132:
180
130
100
70
55
40
CAOV3
piR-26441 mimic
piR-26441 mimic
piR-NC
piR-NC
-
-
+
+
MG132:
YTHDC1 100kDa
GAPDH 36kDa
piR-26441 mimic
piR-26441 mimic
piR-NC
piR-NC
-
-
+
+
MG132:
YTHDC1 100kDa
GAPDH 36kDa
piR-26441 mimic
piR-26441 mimic
piR-NC
piR-NC
-
-
+
+
MG132:
YTHDC1 100kDa
GAPDH 36kDa
180
130
100
70
55
40

## Slide 6
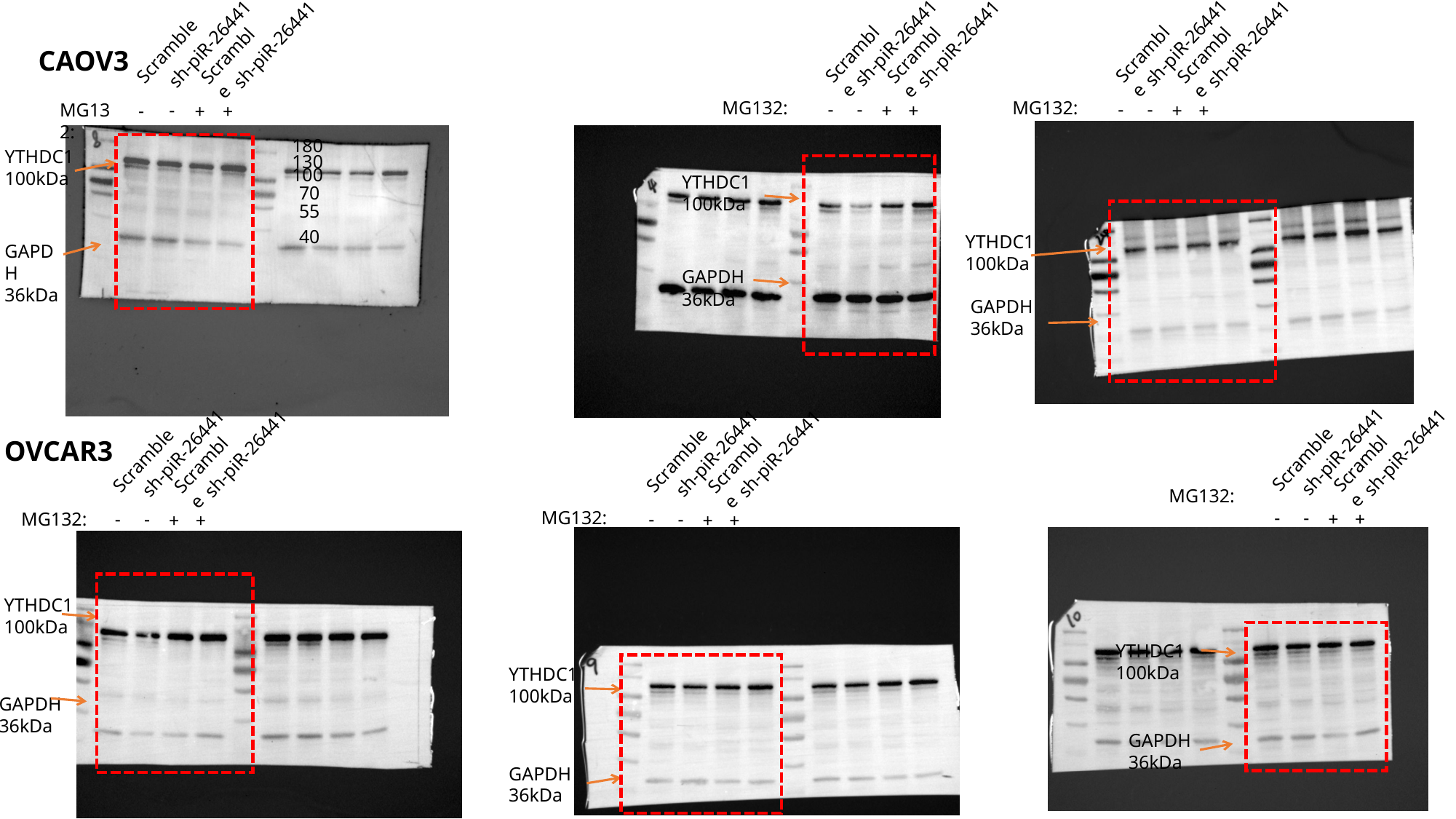

sh-piR-26441
sh-piR-26441
Scramble
Scramble
-
-
+
+
YTHDC1 100kDa
GAPDH 36kDa
MG132:
sh-piR-26441
sh-piR-26441
Scramble
Scramble
-
-
+
+
sh-piR-26441
sh-piR-26441
Scramble
Scramble
-
-
+
+
MG132:
180
130
100
70
55
40
YTHDC1 100kDa
GAPDH 36kDa
CAOV3
MG132:
YTHDC1 100kDa
GAPDH 36kDa
sh-piR-26441
sh-piR-26441
Scramble
Scramble
-
-
+
+
sh-piR-26441
sh-piR-26441
Scramble
Scramble
-
-
+
+
sh-piR-26441
sh-piR-26441
Scramble
Scramble
-
-
+
+
OVCAR3
MG132:
MG132:
MG132:
YTHDC1 100kDa
GAPDH 36kDa
YTHDC1 100kDa
GAPDH 36kDa
YTHDC1 100kDa
GAPDH 36kDa

## Slide 7
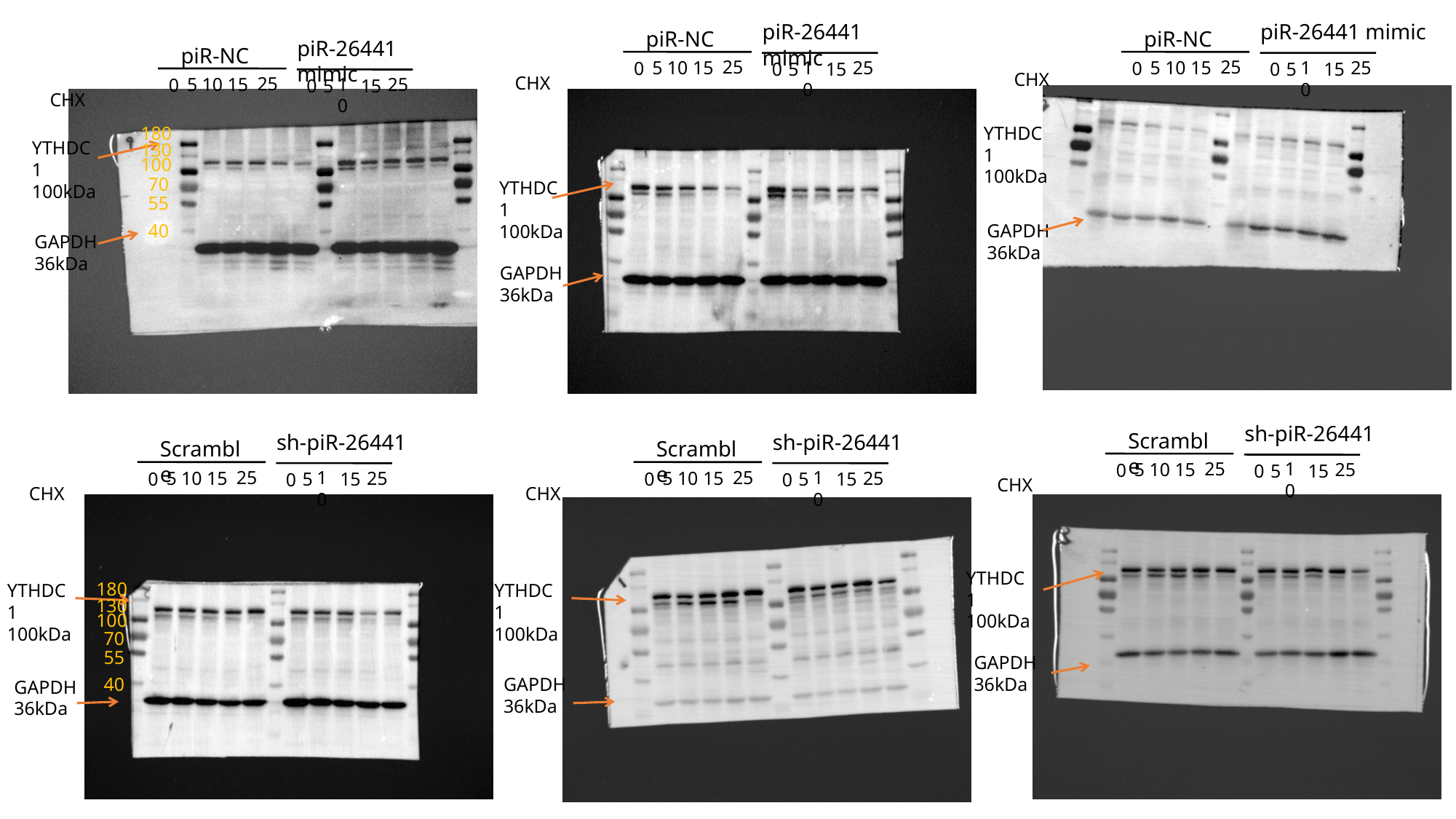

piR-26441 mimic
10
25
5
15
0
piR-NC
25
5
10
15
0
CHX
YTHDC1 100kDa
GAPDH 36kDa
piR-26441 mimic
10
25
5
15
0
piR-NC
25
5
10
15
0
CHX
YTHDC1 100kDa
GAPDH 36kDa
piR-26441 mimic
10
25
5
15
0
piR-NC
25
5
10
15
0
CHX
YTHDC1 100kDa
GAPDH 36kDa
180
130
100
70
55
40
sh-piR-26441
10
25
5
15
0
Scramble
25
5
10
15
0
CHX
YTHDC1 100kDa
GAPDH 36kDa
sh-piR-26441
10
25
5
15
0
Scramble
25
5
10
15
0
CHX
YTHDC1 100kDa
GAPDH 36kDa
sh-piR-26441
10
25
5
15
0
Scramble
25
5
10
15
0
CHX
YTHDC1 100kDa
GAPDH 36kDa
180
130
100
70
55
40

## Slide 8
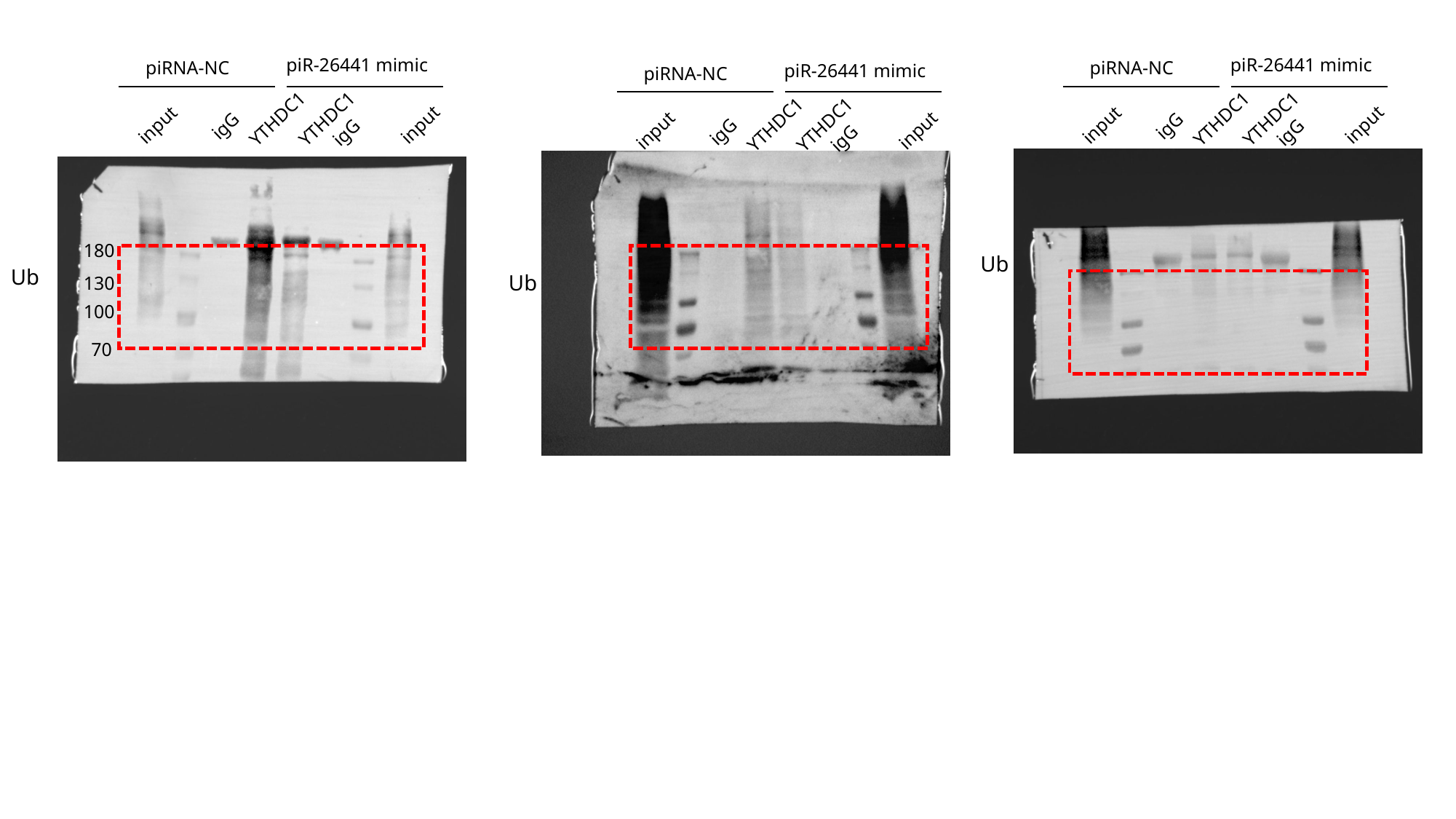

piR-26441 mimic
piRNA-NC
YTHDC1
input
igG
YTHDC1
igG
input
Ub
piR-26441 mimic
piRNA-NC
YTHDC1
input
igG
YTHDC1
igG
input
Ub
piR-26441 mimic
piRNA-NC
YTHDC1
input
igG
YTHDC1
igG
input
Ub
180
130
100
70

## Slide 9
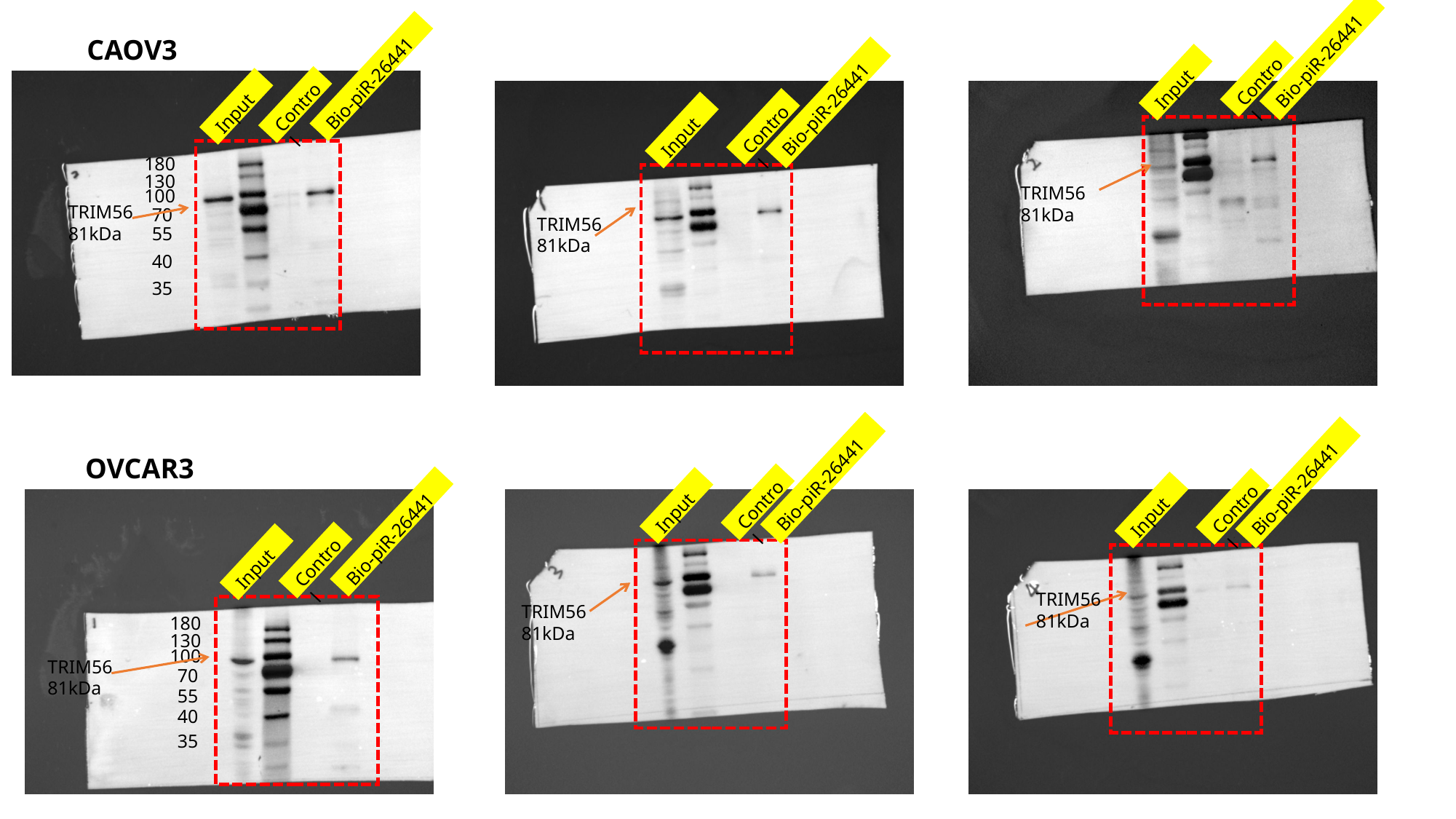

Bio-piR-26441
Control
Input
Bio-piR-26441
Control
Input
Bio-piR-26441
Control
Input
CAOV3
180
130
100
70
55
40
35
TRIM56
81kDa
TRIM56
81kDa
TRIM56
81kDa
Bio-piR-26441
Control
Input
Bio-piR-26441
Control
Input
OVCAR3
Bio-piR-26441
Control
Input
TRIM56
81kDa
TRIM56
81kDa
180
130
100
70
55
40
35
TRIM56
81kDa

## Slide 10
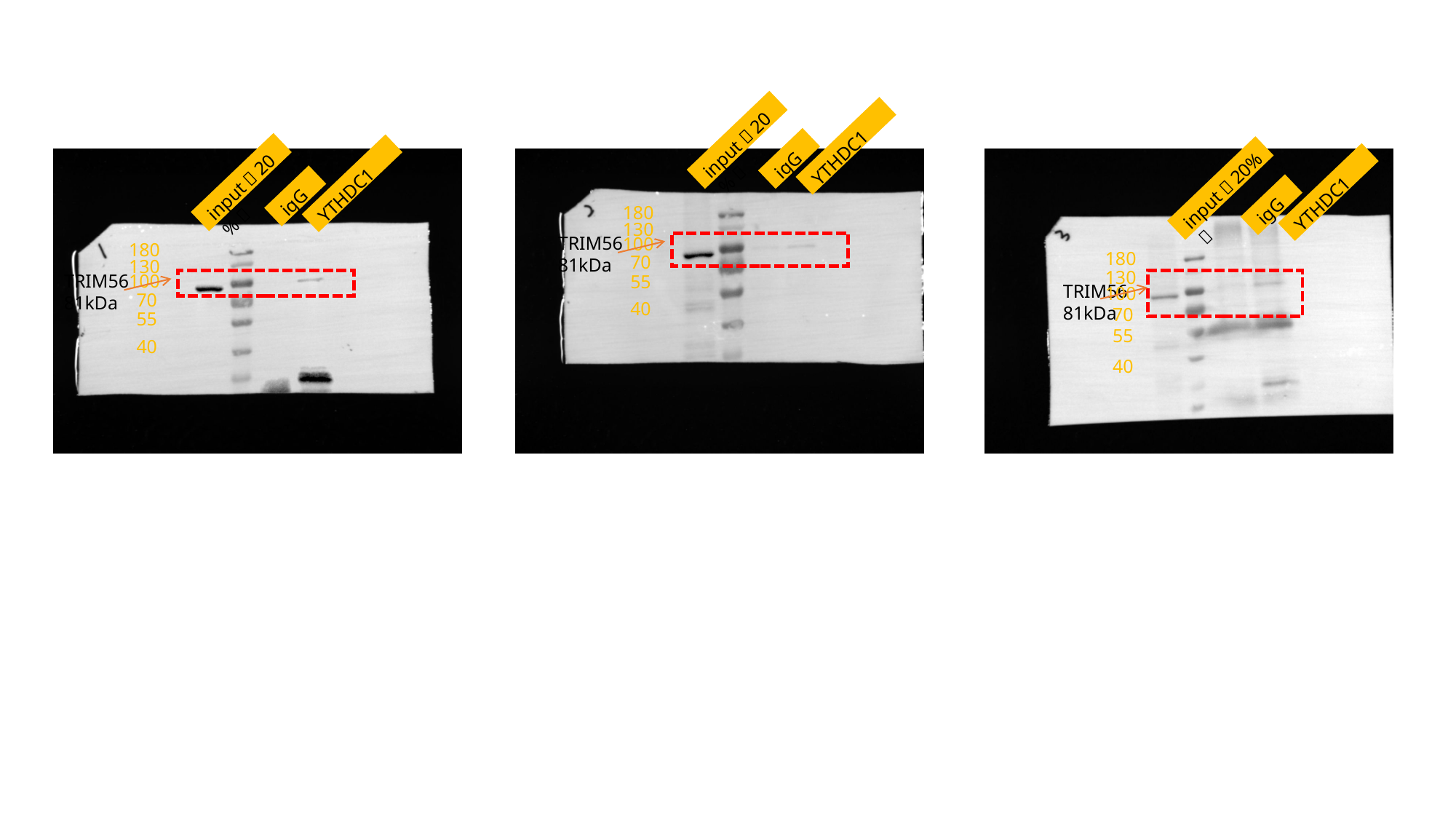

input（20%）
YTHDC1
igG
180
130
100
70
55
40
TRIM56
81kDa
input（20%）
YTHDC1
igG
180
130
100
70
55
40
TRIM56
81kDa
input（20%）
YTHDC1
igG
180
130
100
70
55
40
TRIM56
81kDa

## Slide 11
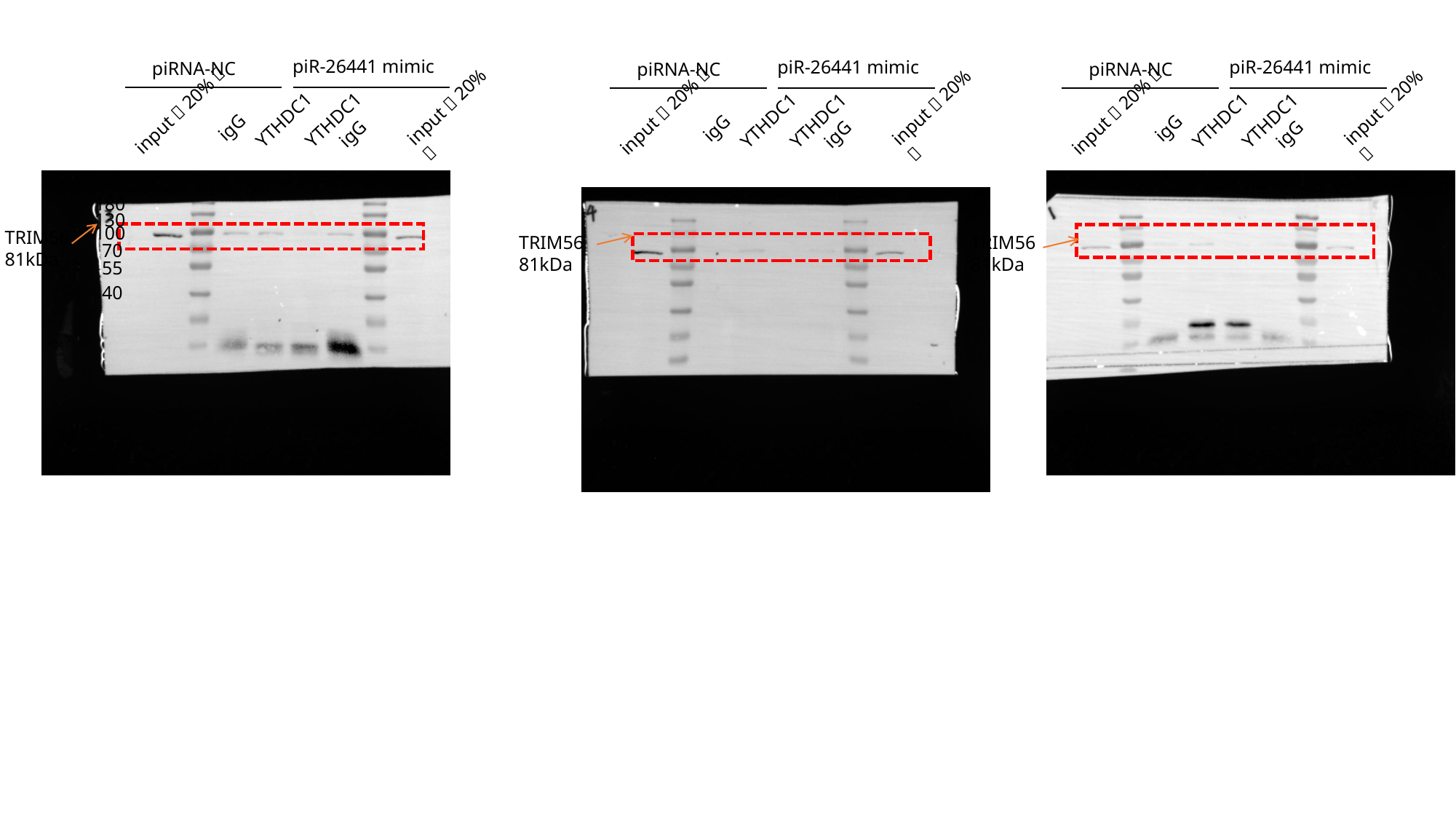

piR-26441 mimic
piRNA-NC
input（20%）
input（20%）
YTHDC1
igG
YTHDC1
igG
TRIM56
81kDa
piR-26441 mimic
piRNA-NC
input（20%）
input（20%）
YTHDC1
igG
YTHDC1
igG
TRIM56
81kDa
piR-26441 mimic
piRNA-NC
input（20%）
input（20%）
YTHDC1
igG
YTHDC1
igG
TRIM56
81kDa
180
130
100
70
55
40

## Slide 12
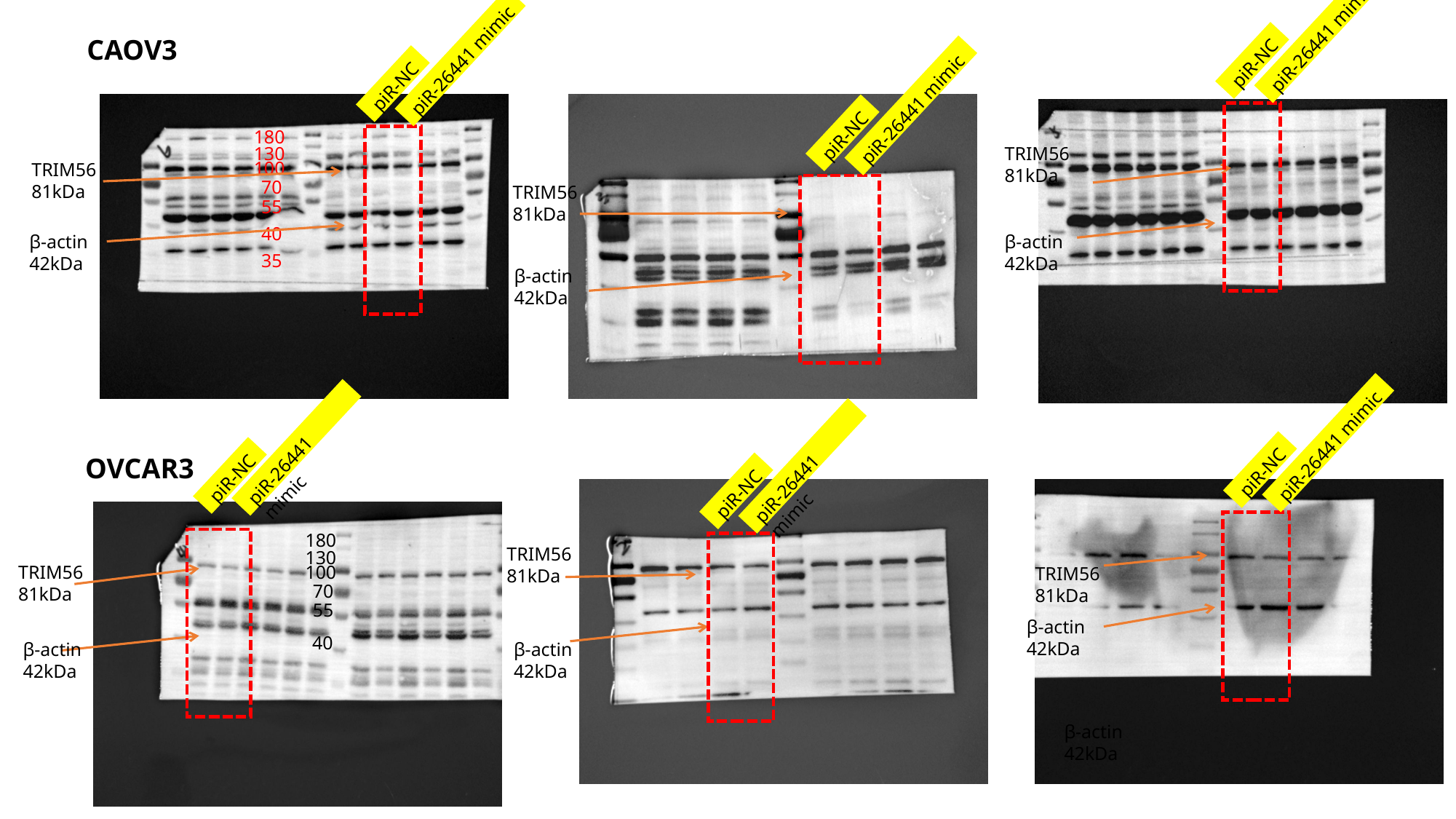

piR-26441 mimic
piR-NC
piR-26441 mimic
piR-NC
β-actin
42kDa
piR-26441 mimic
piR-NC
CAOV3
180
130
100
70
55
40
35
TRIM56
81kDa
TRIM56
81kDa
TRIM56
81kDa
β-actin
42kDa
β-actin
42kDa
piR-26441 mimic
piR-NC
piR-26441 mimic
piR-NC
180
130
100
70
55
40
piR-26441 mimic
piR-NC
OVCAR3
TRIM56
81kDa
β-actin
42kDa
TRIM56
81kDa
TRIM56
81kDa
β-actin
42kDa
β-actin
42kDa
β-actin
42kDa

## Slide 13
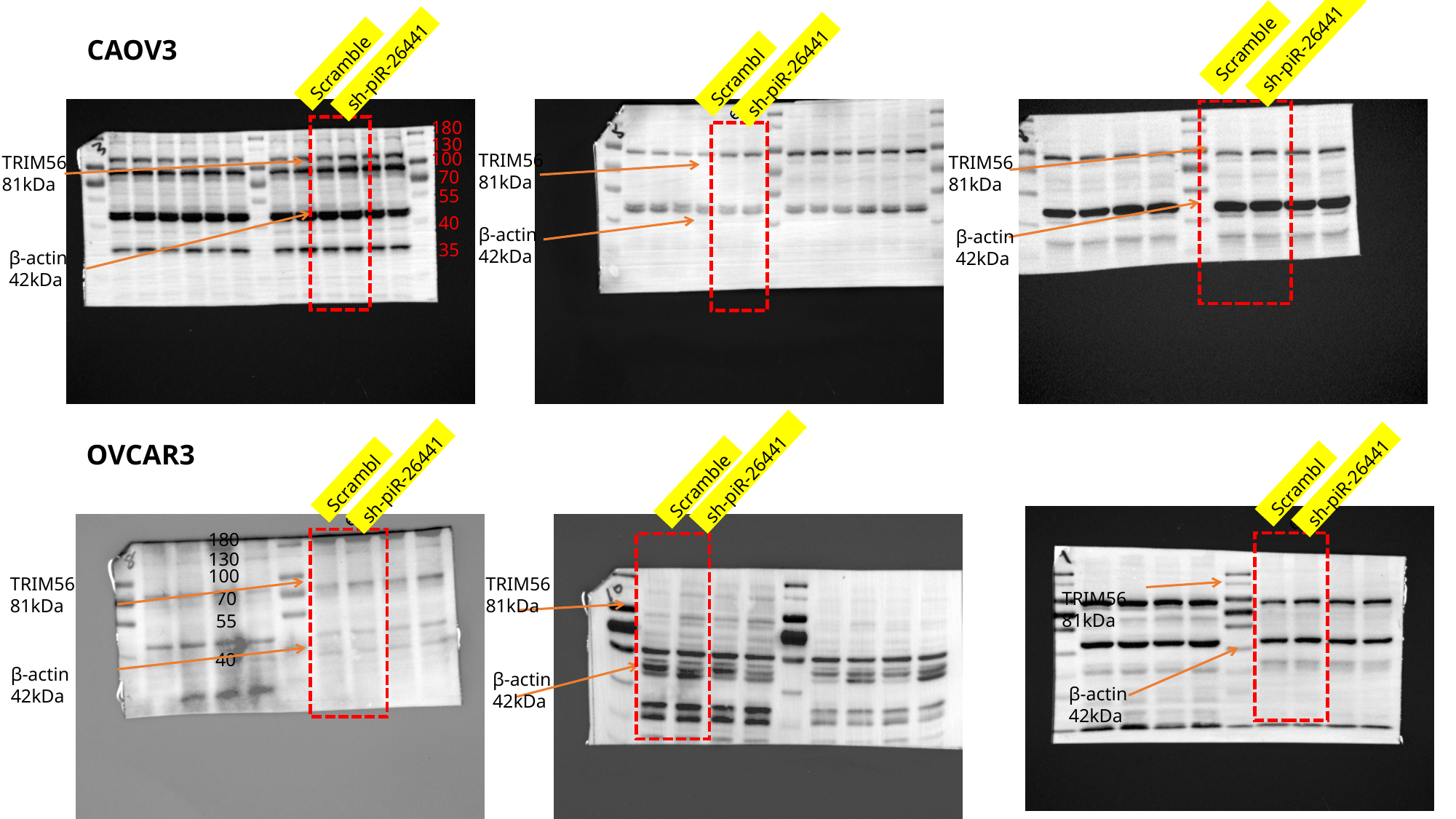

sh-piR-26441
Scramble
Scramble
sh-piR-26441
180
130
100
70
55
40
35
TRIM56
81kDa
β-actin
42kDa
sh-piR-26441
Scramble
CAOV3
TRIM56
81kDa
β-actin
42kDa
TRIM56
81kDa
β-actin
42kDa
sh-piR-26441
Scramble
sh-piR-26441
Scramble
sh-piR-26441
Scramble
OVCAR3
180
130
100
70
55
40
TRIM56
81kDa
β-actin
42kDa
TRIM56
81kDa
β-actin
42kDa
TRIM56
81kDa
β-actin
42kDa

## Slide 14
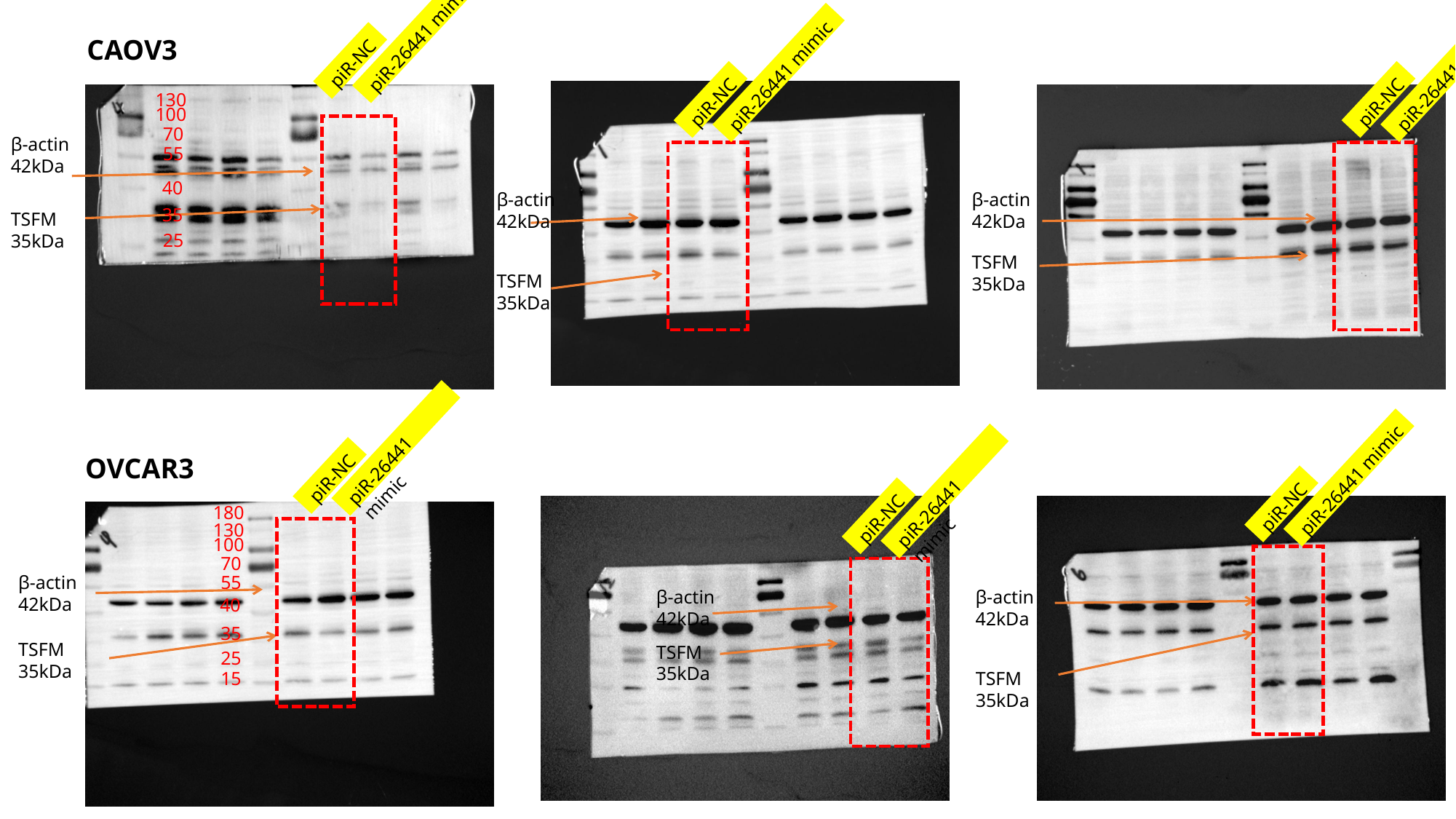

piR-26441 mimic
piR-NC
piR-26441 mimic
piR-NC
piR-26441 mimic
piR-NC
CAOV3
130
100
70
55
40
35
25
β-actin
42kDa
TSFM
35kDa
β-actin
42kDa
TSFM
35kDa
β-actin
42kDa
TSFM
35kDa
piR-26441 mimic
piR-NC
piR-26441 mimic
piR-NC
piR-26441 mimic
piR-NC
OVCAR3
180
130
100
70
55
40
35
25
15
β-actin
42kDa
TSFM
35kDa
β-actin
42kDa
TSFM
35kDa
β-actin
42kDa
TSFM
35kDa

## Slide 15
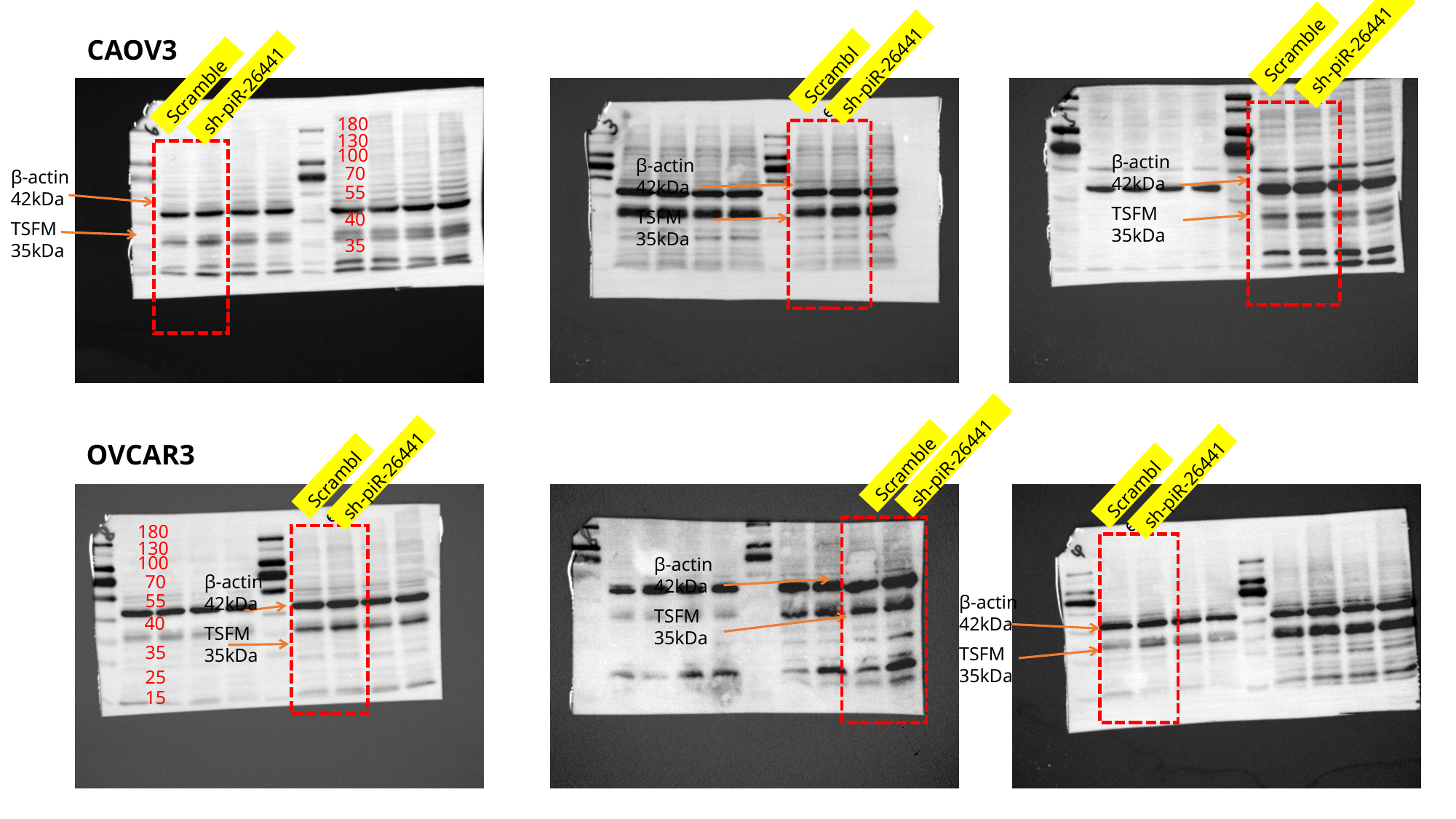

sh-piR-26441
Scramble
sh-piR-26441
Scramble
Scramble
sh-piR-26441
CAOV3
180
130
100
70
55
40
35
β-actin
42kDa
TSFM
35kDa
β-actin
42kDa
TSFM
35kDa
β-actin
42kDa
TSFM
35kDa
sh-piR-26441
Scramble
sh-piR-26441
Scramble
sh-piR-26441
Scramble
OVCAR3
180
130
100
70
55
40
35
25
15
β-actin
42kDa
TSFM
35kDa
β-actin
42kDa
TSFM
35kDa
β-actin
42kDa
TSFM
35kDa

## Slide 16
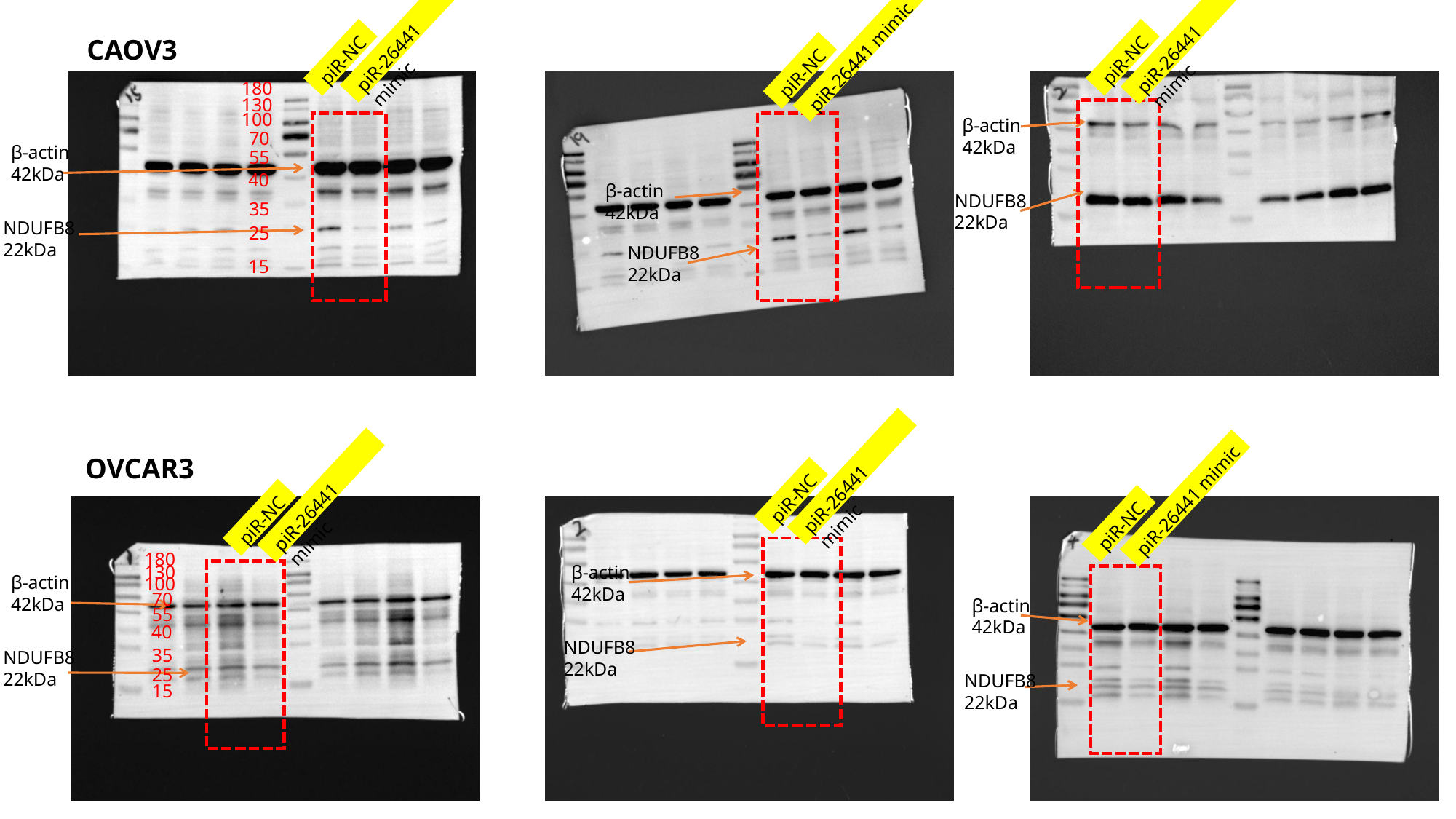

piR-26441 mimic
piR-NC
piR-26441 mimic
piR-NC
piR-26441 mimic
piR-NC
CAOV3
180
130
100
70
55
40
35
25
15
β-actin
42kDa
NDUFB822kDa
β-actin
42kDa
NDUFB822kDa
β-actin
42kDa
NDUFB822kDa
piR-26441 mimic
piR-NC
piR-26441 mimic
piR-NC
piR-26441 mimic
piR-NC
OVCAR3
180
130
100
70
55
40
35
25
15
β-actin
42kDa
NDUFB822kDa
β-actin
42kDa
NDUFB822kDa
β-actin
42kDa
NDUFB822kDa

## Slide 17
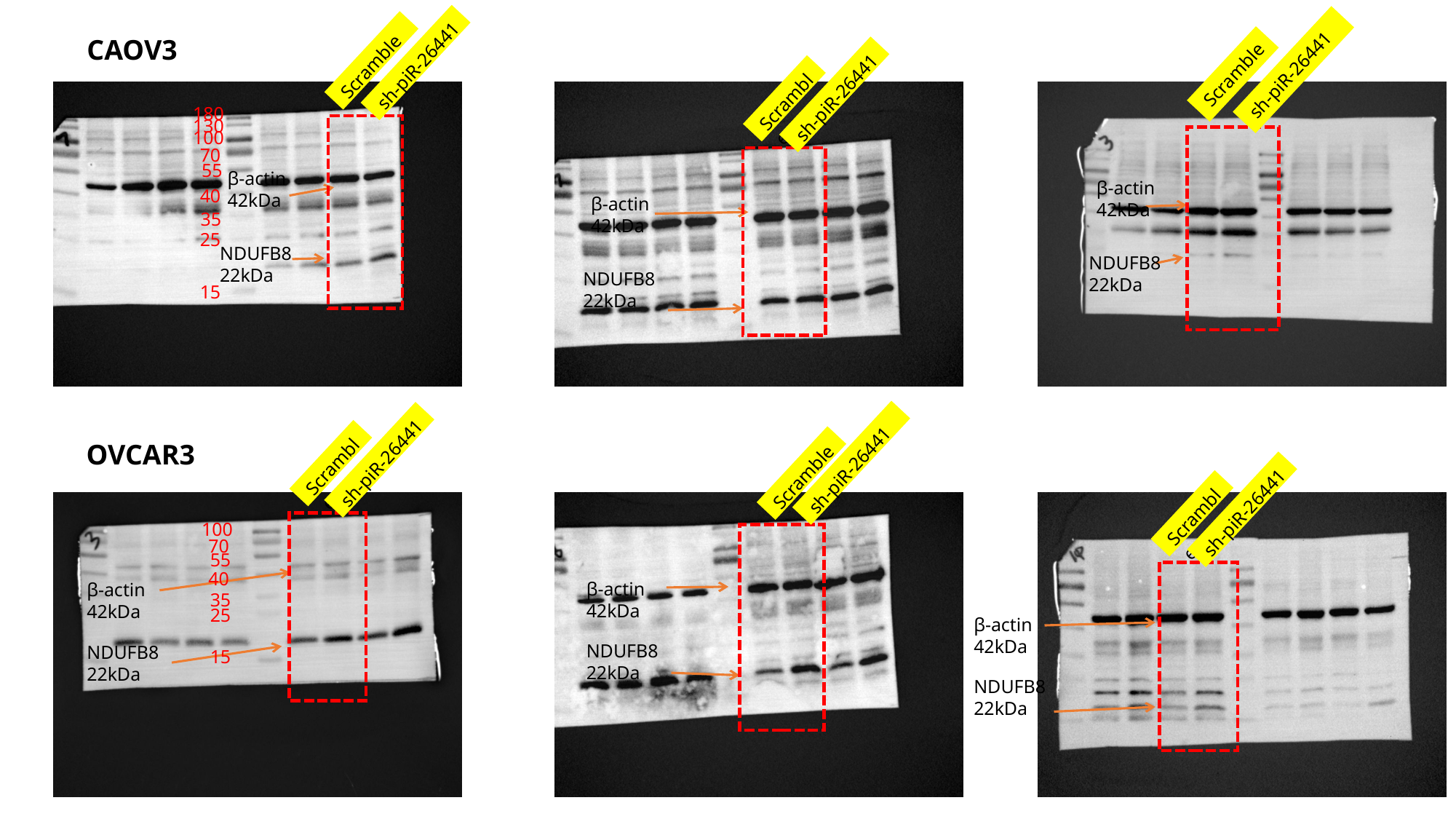

Scramble
sh-piR-26441
sh-piR-26441
Scramble
CAOV3
sh-piR-26441
Scramble
180
130
100
70
55
40
35
25
15
β-actin
42kDa
NDUFB822kDa
β-actin
42kDa
NDUFB822kDa
β-actin
42kDa
NDUFB822kDa
sh-piR-26441
Scramble
sh-piR-26441
Scramble
OVCAR3
sh-piR-26441
Scramble
100
70
55
40
35
25
15
β-actin
42kDa
NDUFB822kDa
β-actin
42kDa
NDUFB822kDa
β-actin
42kDa
NDUFB822kDa

## Slide 18
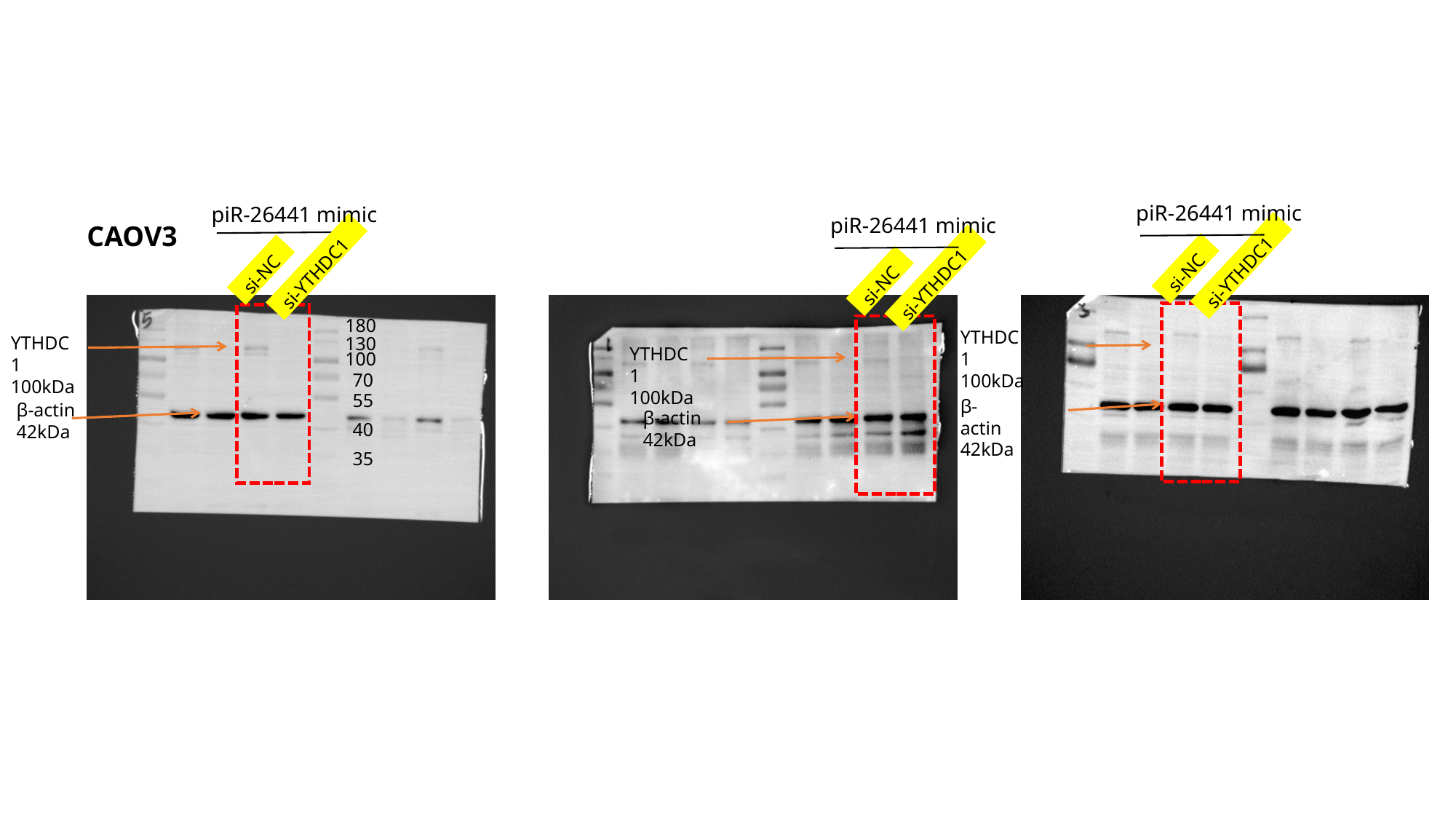

piR-26441 mimic
si-YTHDC1
si-NC
YTHDC1 100kDa
β-actin
42kDa
piR-26441 mimic
si-YTHDC1
si-NC
piR-26441 mimic
si-YTHDC1
si-NC
YTHDC1 100kDa
β-actin
42kDa
CAOV3
180
130
100
70
55
40
35
YTHDC1 100kDa
β-actin
42kDa

## Slide 19
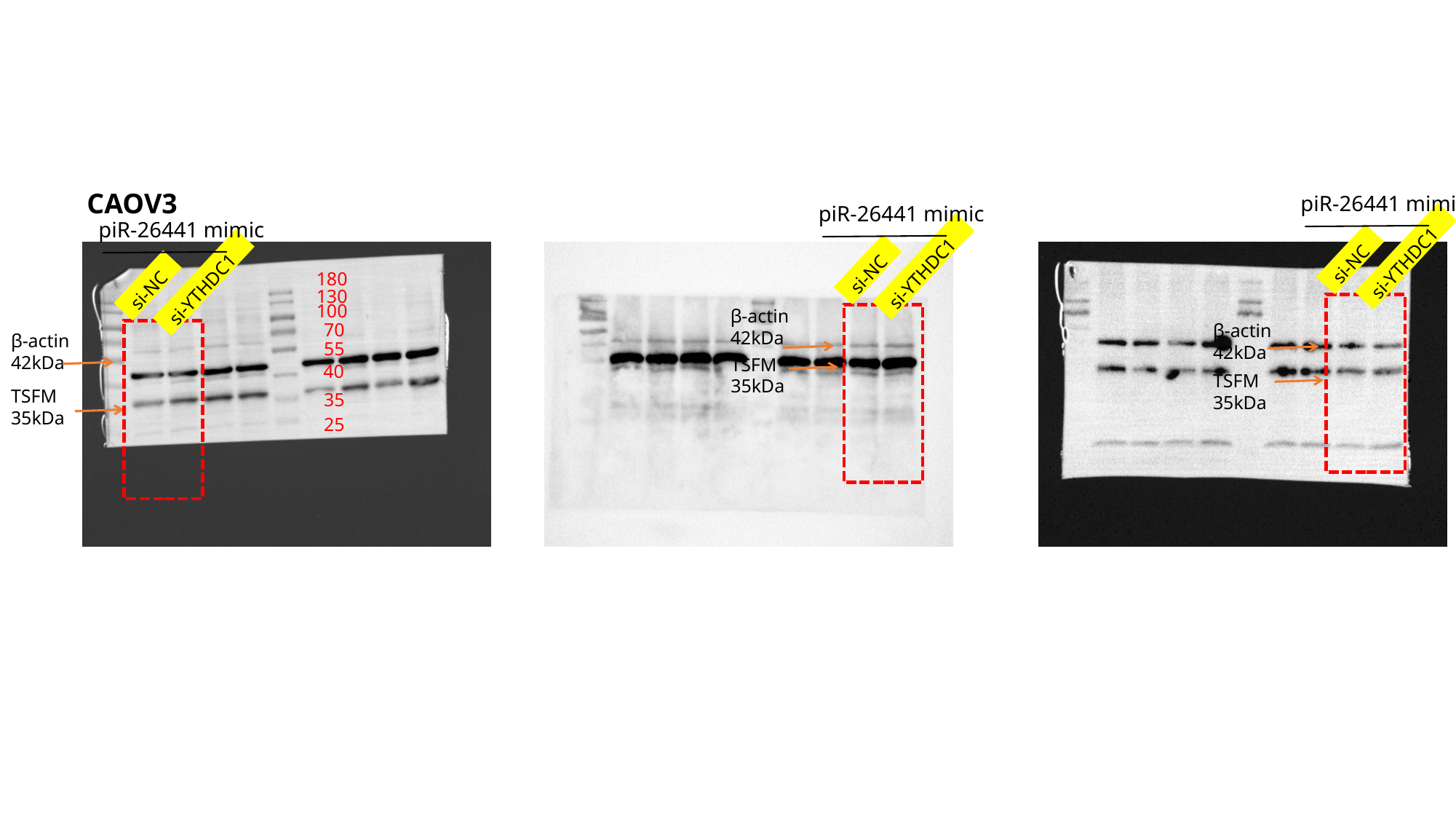

CAOV3
piR-26441 mimic
si-YTHDC1
si-NC
β-actin
42kDa
TSFM
35kDa
piR-26441 mimic
si-YTHDC1
si-NC
β-actin
42kDa
TSFM
35kDa
piR-26441 mimic
si-YTHDC1
si-NC
β-actin
42kDa
TSFM
35kDa
180
130
100
70
55
40
35
25

## Slide 20
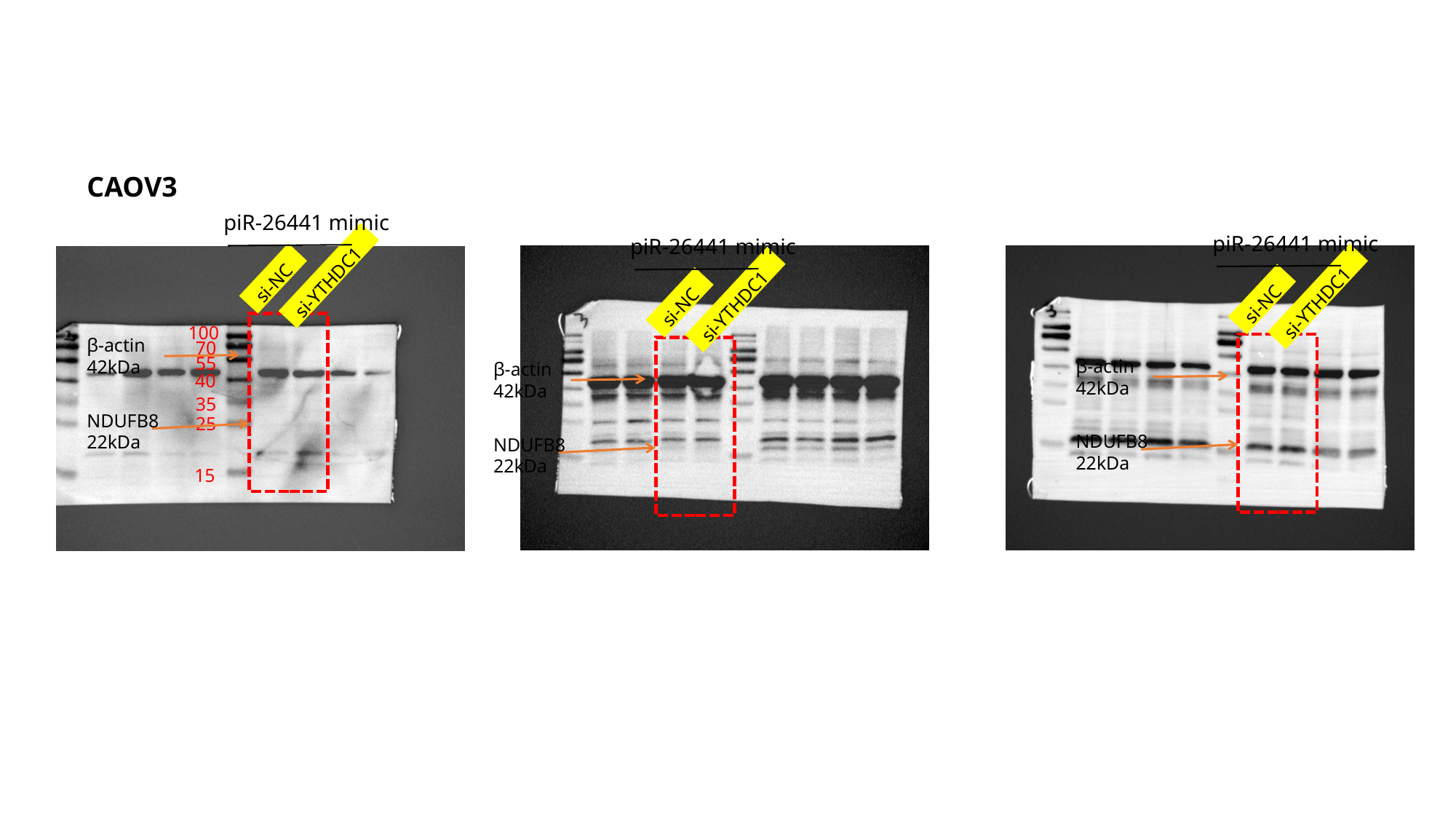

CAOV3
piR-26441 mimic
si-YTHDC1
si-NC
β-actin
42kDa
NDUFB822kDa
piR-26441 mimic
si-YTHDC1
si-NC
β-actin
42kDa
NDUFB822kDa
piR-26441 mimic
si-YTHDC1
si-NC
β-actin
42kDa
NDUFB822kDa
100
70
55
40
35
25
15

## Slide 21
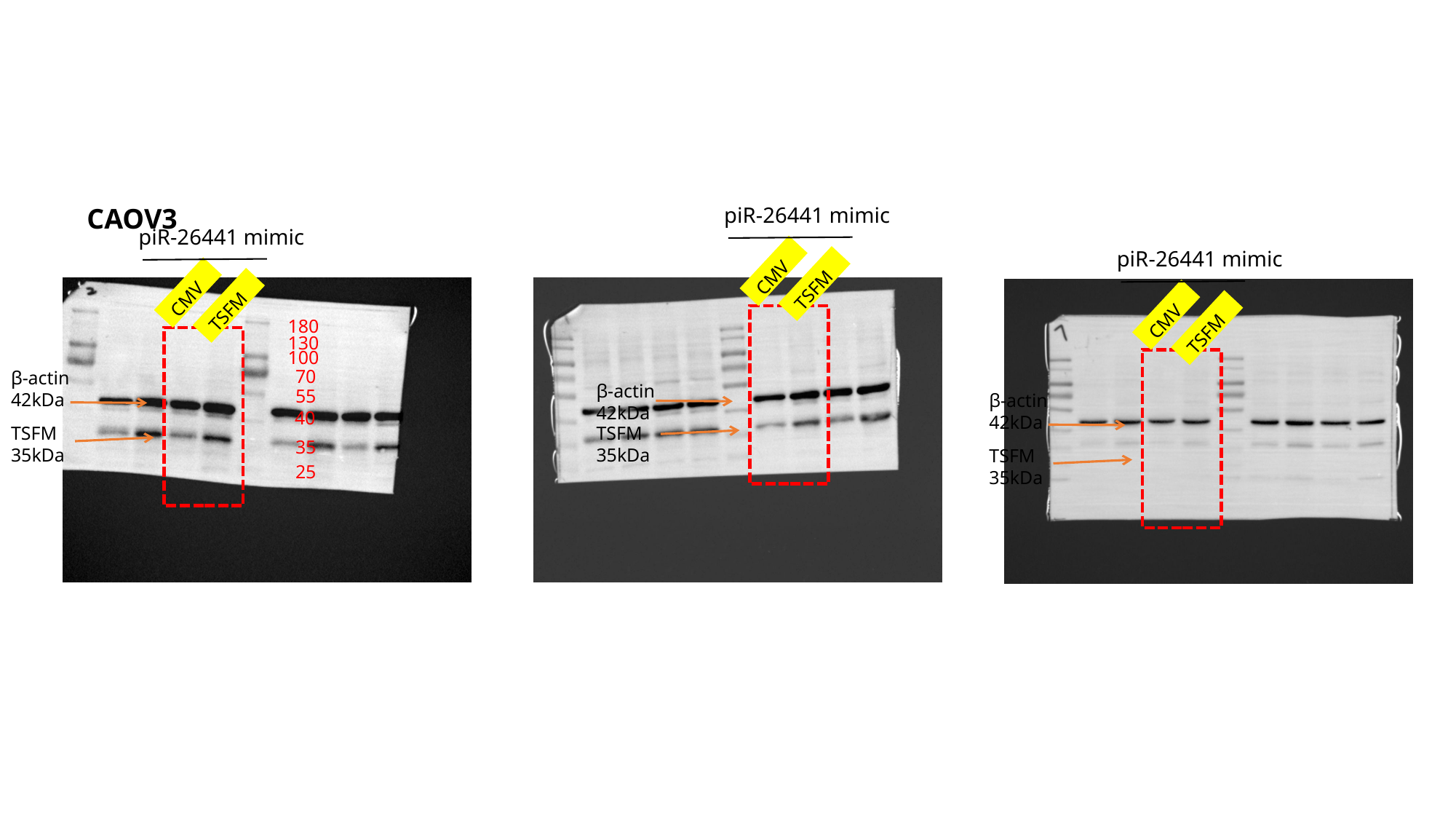

CAOV3
piR-26441 mimic
CMV
TSFM
β-actin
42kDa
TSFM
35kDa
piR-26441 mimic
CMV
TSFM
β-actin
42kDa
TSFM
35kDa
piR-26441 mimic
CMV
TSFM
β-actin
42kDa
TSFM
35kDa
180
130
100
70
55
40
35
25

## Slide 22
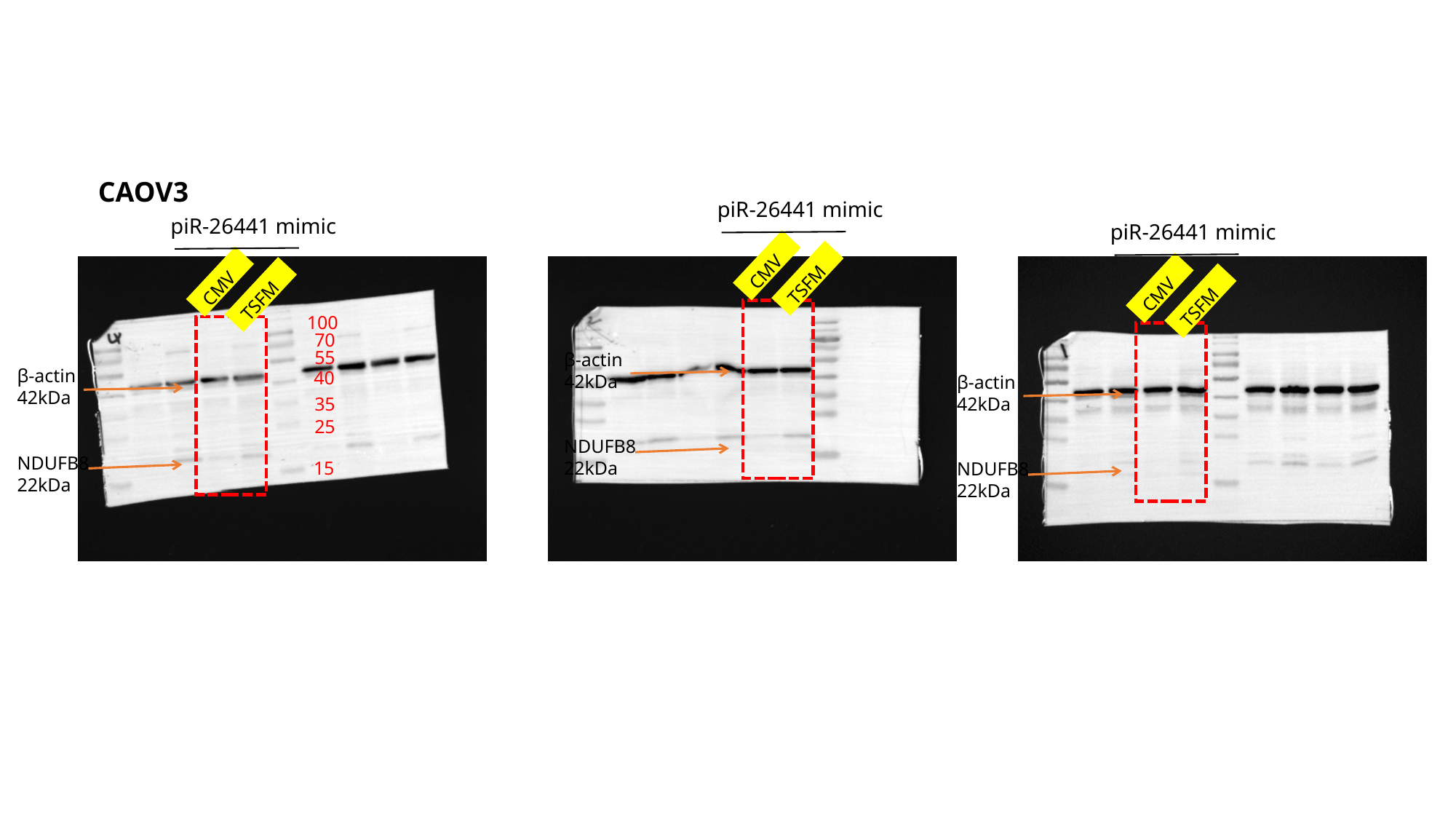

CAOV3
piR-26441 mimic
CMV
TSFM
β-actin
42kDa
NDUFB822kDa
piR-26441 mimic
CMV
TSFM
β-actin
42kDa
NDUFB822kDa
piR-26441 mimic
CMV
TSFM
β-actin
42kDa
NDUFB822kDa
100
70
55
40
35
25
15

## Slide 23
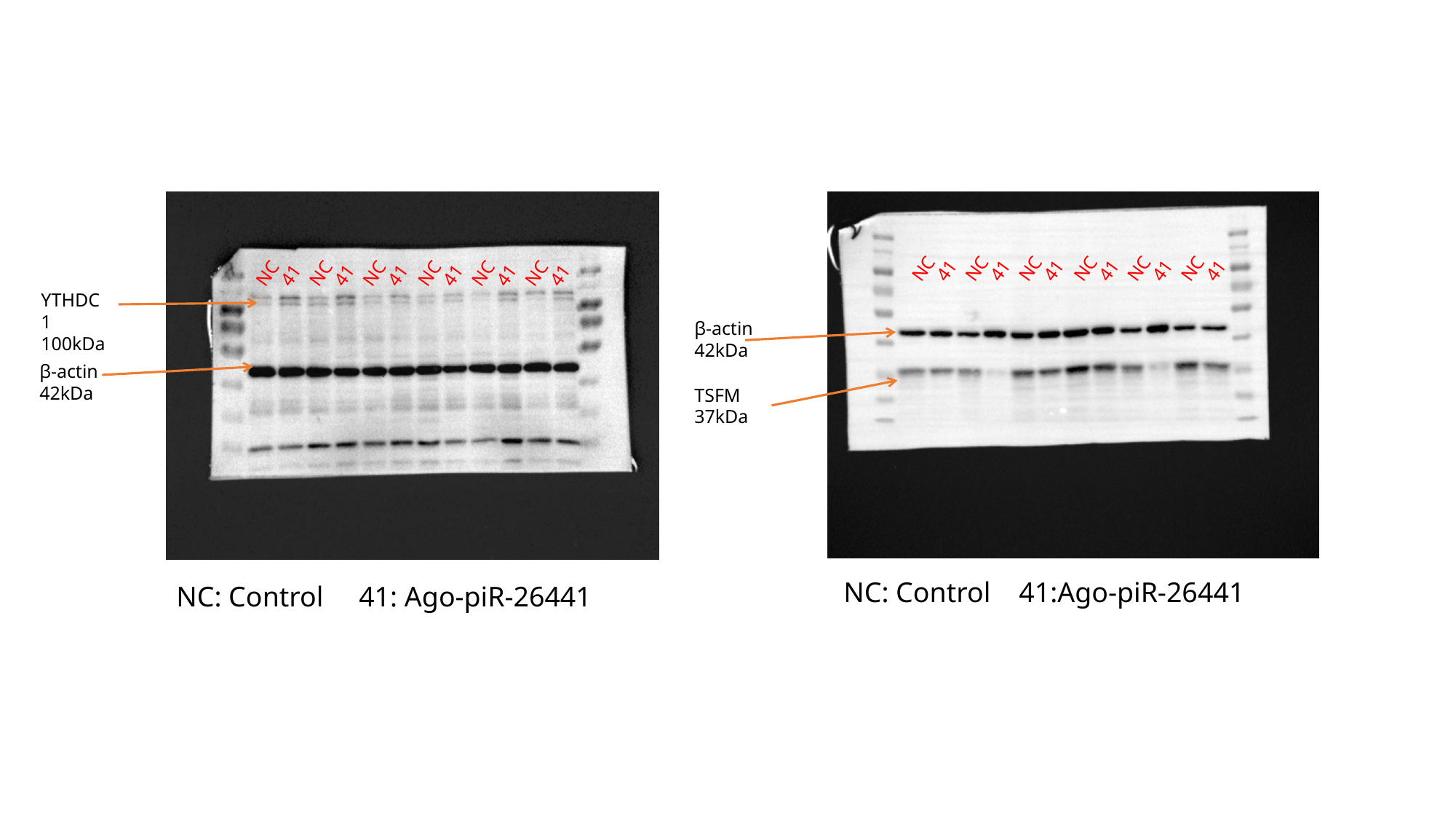

NC
41
NC
41
NC
41
NC
41
NC
41
NC
41
YTHDC1 100kDa
β-actin
42kDa
NC: Control 41: Ago-piR-26441
NC
41
NC
41
NC
41
NC
41
NC
41
NC
41
β-actin
42kDa
TSFM 37kDa
NC: Control 41:Ago-piR-26441
